# Reactive Oxygen Species-Dependent Innate Immune Mechanisms Control Methicillin-Resistant Staphylococcus aureus Virulence in the *Drosophila* Larval Model

**DOI:** 10.1128/mBio.00276-21

**Published:** 2021-06-15

**Authors:** Elodie Ramond, Anne Jamet, Xiongqi Ding, Daniel Euphrasie, Clémence Bouvier, Louison Lallemant, Xiangyan He, Laurence Arbibe, Mathieu Coureuil, Alain Charbit

**Affiliations:** a Université de Paris, Paris, France; b INSERM U1151, CNRS UMR 8253, Institut Necker-Enfants Malades, Paris, France; c Cell Imaging Core Facility, Structure Fédérative de Recherche Necker, INSERM US24/CNRS UMS3633, Paris, France; d Imagine, Université de Paris, Paris, France; University of Melbourne; Institut Pasteur

**Keywords:** *Staphylococcus aureus*, *Drosophila melanogaster*, intestinal infection, virulence, catalase, Duox, gastrointestinal infection

## Abstract

Antibiotic-resistant Staphylococcus aureus strains constitute a major public health concern worldwide and are responsible for both health care- and community-associated infections. Here, we establish a robust and easy-to-implement model of oral S. aureus infection using Drosophila melanogaster larvae that allowed us to follow the fate of S. aureus at the whole-organism level as well as the host immune responses. Our study demonstrates that S. aureus infection triggers H_2_O_2_ production by the host via the Duox enzyme, thereby promoting antimicrobial peptide production through activation of the Toll pathway. Staphylococcal catalase mediates H_2_O_2_ neutralization, which not only promotes S. aureus survival but also minimizes the host antimicrobial response, hence reducing bacterial clearance *in vivo*. We show that while catalase expression is regulated *in vitro* by the accessory gene regulatory system (Agr) and the general stress response regulator sigma B (SigB), it no longer depends on these two master regulators *in vivo*. Finally, we confirm the versatility of this model by demonstrating the colonization and host stimulation capabilities of S. aureus strains belonging to different sequence types (CC8 and CC5) as well as of two other bacterial pathogens, Salmonella enterica serovar Typhimurium and Shigella flexneri. Thus, the *Drosophila* larva can be a general model to follow *in vivo* the innate host immune responses triggered during infection by human pathogens.

## INTRODUCTION

Staphylococcus aureus is a facultative aerobic Gram-positive bacterium that behaves as a commensal microorganism (up to 30% of the healthy human population carries S. aureus through nasal, skin, and intestinal colonization) or as a pathogen causing a wide range of infections in humans and in wild and companion animals (1–3). The emergence and diffusion of methicillin-resistant S. aureus (MRSA) clones that express numerous virulence factors, including toxins and adhesins increasing their toxicity and colonization capacities, are a major public health issue. Expression of these numerous virulence factors are correlated with severe symptoms among previously healthy colonized individuals (4–6). During infection, S. aureus must face host innate immunity, i.e., phagocyte-mediated elimination via oxidative stress (by macrophages and neutrophils) and antimicrobial peptides secretion (7). S. aureus undergoes both endogenous oxidative stress (notably caused by incomplete aerobic respiration) and exogenous host-induced oxidative stress aimed at killing the bacteria (8, 9). Host reactive oxygen species (ROS) are secreted by the Nox/Duox NADPH (NAD phosphate) oxidases. In mammals, Nox and Duox families are composed, respectively, of five Noxs (Nox1 to Nox5) and two Duoxs (Duox1 and Duox2) members. Nox1 and Duox2 are found in the gastrointestinal tract, and Nox2 was identified in phagocytic cells (10–12), while other enzymes are expressed in other tissues, such as airway epithelium, kidneys, endothelial cells, etc. (13, 14). Duox enzymes generate hydrogen peroxide (H_2_O_2_), whereas Nox enzymes catalyze superoxide production (O_2_·^–^). Of note, mitochondria from phagocytic cells also contribute in ROS production to counteract infection (15). To neutralize the deleterious effects of ROS, S. aureus USA300 expresses multiple direct detoxifying enzymes, including (i) the two superoxide dismutases SodA and SodM, which convert the superoxide anion O_2_·^–^ to H_2_O_2_ and O_2_; (ii) the H_2_O_2_-detoxifying catalase KatA and the alkyl hydroperoxide reductase AhpC, which, respectively, quench high and low H_2_O_2_ concentrations; and (iii) the two glutathione peroxidases GpxA1 and GpxA2, which also catalyze H_2_O_2_ reduction into water through glutathione (GSH) oxidation (16). It is known that *sodA* transcription depends on two sigma factors, the first being sigma A (σ^A^)-type promoters and the second being the alternative stress-activated sigma B factor (σ^B^), which represses *sodA*; *sodM* is repressed solely by σ^B^ (17). More recently, it was shown that *sodA* and *sodM* expression is repressed by the *msaABCR* operon (18). In contrast, the *katA* gene is upregulated through the ferric uptake regulator Fur under iron-rich conditions (19). Under low-iron or manganese-rich conditions, the metal-dependent transcription factor PerR inhibits Fur, resulting in the downregulation of *katA*. (20).

Although most *in vivo* studies rely on the mouse model, mechanistic and genetic analyses can be performed with powerful alternative animal models, such as Drosophila melanogaster (fly) or Danio rerio (zebrafish). Of note, flies share many similarities with humans with respect to gastrointestinal anatomy and physiology (21), while the zebrafish model displays several disadvantages for intestinal infection research, mainly due to limited pH variations (the pH remains around 7.5) (22, 23). Furthermore, the *Drosophila* gut microbiota, which includes only a few bacterial species (mainly from the two bacterial families *Lactobacillaceae* and *Acetobacteraceae*) (24, 25), is closer to the human microbiota than it is to that of zebrafish, which is colonized mainly by the class gammaproteobacteria and more specifically by the *Aeromonas* genus (26). The D. melanogaster intestine consists of a simple ciliated epithelium layer surrounded by a muscle layer (27) that has the ability to develop a proper innate immune response to intestinal bacteria, including tolerating mechanisms for beneficial microbiota (28), similarly to mammals (29). The peritrophic matrix establishes a physical barrier that isolates pathogenic bacteria and their toxins from the epithelium layer (30).

When infected, the *Drosophila* intestinal epithelium, at all stages, can generate a robust antimicrobial response. On one hand, it involves the secretion of antimicrobial peptides (AMPs). They are produced (i) either upon Toll pathway activation, similarly to the MyD88 Toll-like receptor pathway in mammals, reacting to Gram-positive bacteria and fungi, (ii) or upon immune deficiency (IMD) pathway activation, which shares many similarities with the tumor necrosis factor (TNF) cascade, reacting to Gram-negative bacteria (31, 32). In addition to activating these two pathways, the fly can clear pathogenic bacteria by activating the production of microbicidal reactive oxygen species (ROS) via the Duox pathway (33). Several studies showed that the *Drosophila* model recapitulates many aspects of the human intestinal pathologies (34) and has already allowed the successful evaluation of the harmfulness of human pathogens such as Mycobacterium tuberculosis (35), Listeria monocytogenes (36), Vibrio cholerae (37), Francisella tularensis (38), Pseudomonas aeruginosa (39), and Yersinia pestis (40).

The lack of a satisfactory *in vivo* model to study S. aureus virulence prompted us to develop an alternative D. melanogaster model that mimics mammalian immune responses to bacterial infections. To date, several S. aureus infection models have been assessed with adult flies. Systemic infections (via pricking in the thorax) result in different outcomes that depend on the dose used and the strain tested, while oral infections showed a limited, or no, infection cost for the host (41–46). Specifically, of the previously published research papers presenting models of intestinal infection in *Drosophila*, none used the epidemic methicillin-resistant S. aureus USA300 strain. Thus far, S. aureus USA300 virulence has been assessed only by septic injury in flies, leading to animal death in a more severe way than with poorly virulent strains, i.e., S. aureus NCTC8325 RN1 and CMRSA6 or the colonization strain M92 (43–46).

In this work, we took advantage of the *Drosophila* larval stage, where animals feed continuously and massively, to establish a new infection model based on the virulence of S. aureus USA300. We also demonstrate the colonization capabilities of Salmonella enterica serovar Typhimurium and Shigella flexneri, suggesting that *Drosophila* larvae can serve as a general model for studying multiple human pathogens.

## RESULTS

### D. melanogaster larvae as a new model to study S. aureus USA300 virulence.

**FIG 1:**
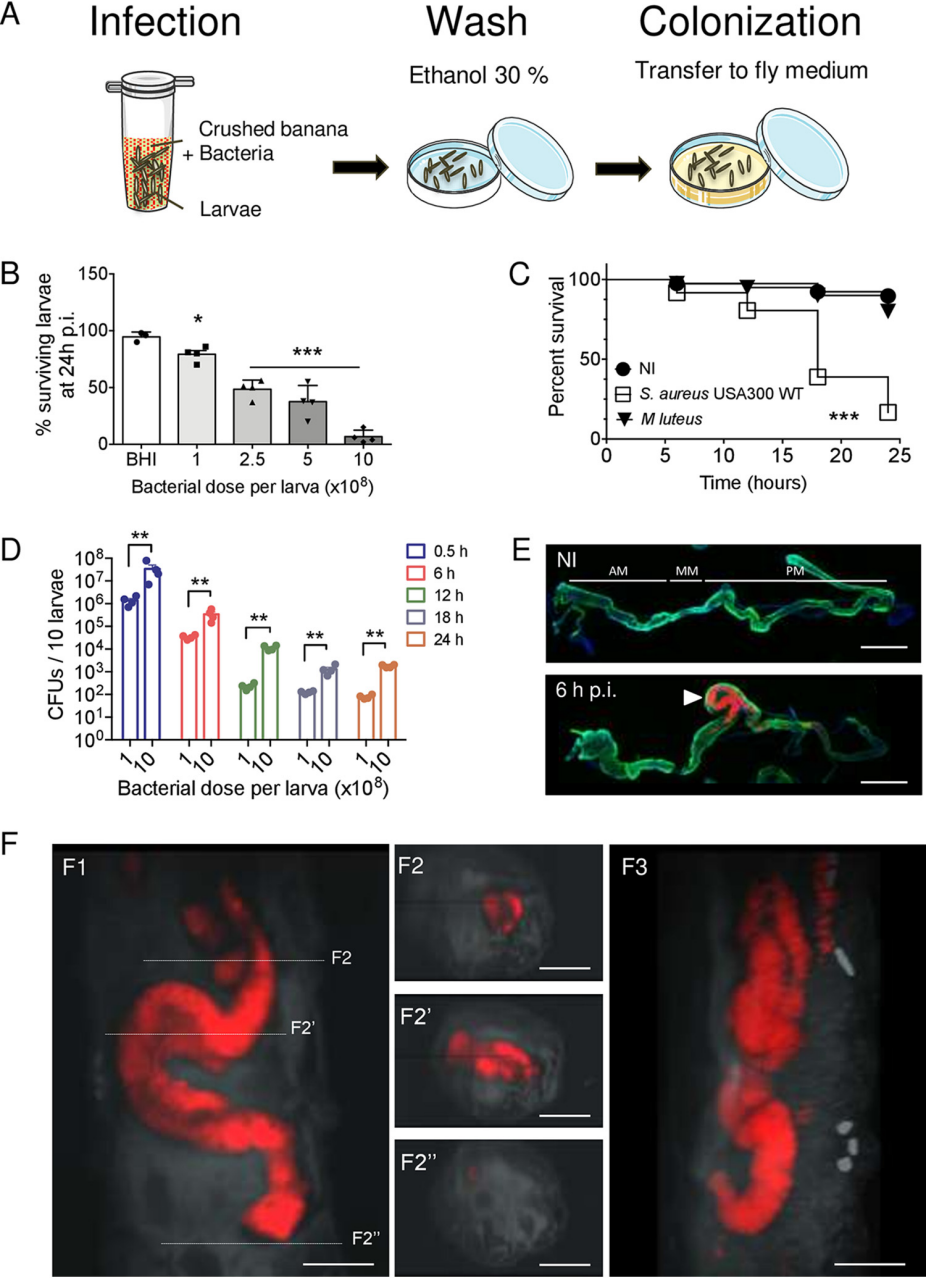
The D. melanogaster larva, a model to study S. aureus USA300 virulence. (A) Mid-L3 larvae were placed in a microcentrifuge tube with 100 μl of crushed banana and 100 μl of bacteria for 30 min. Then larvae were briefly washed with 30% ethanol and transferred to a petri dish with fresh fly medium until further processing. (B) Survival of *w^1118^* D. melanogaster larvae following 30 min of oral infection with wild-type *S aureus* USA300 at the indicated infectious doses. Animals were checked 24 h after infection. Data are means ± SEM (*n* = 3) with 20 animals/point. One-way ANOVA and multiple-comparison tests were performed between infected animals and BHI agar-treated (noninfected) animals (*, *P < *0.05; ***, *P < *0.001). (C) Survival of *w^1118^* D. melanogaster larvae upon 30 min of oral infection with 10 × 10^8^ *S aureus* USA300 WT bacteria and the nonpathogenic entomopathogen Micrococcus luteus. Animals were monitored at 0, 6, 12, 18, and 24 h after infection. Seventy animals from 3 independent experiments were used. The Kaplan-Meier test was applied to the whole group (***, *P < *0.001). NI, noninfected larvae. (D) *w^1118^* D. melanogaster larvae were orally infected for 30 min with 1 × 10^8^ and 10 × 10^8^ chloramphenicol-resistant *S aureus* USA300 WT bacteria (carrying the pRN11 plasmid)/larva. Bacterial counts (CFU) in the gut were determined at 0.5, 6, 12, 18, and 24 h p.i. in live larvae. Tissues were homogenized in DPBS, serially diluted, and plated on BHI agar supplemented with chloramphenicol (10 μg · ml^−1^). Data are means ± SEM (*n* = 3). One-way ANOVA and then multiple-comparison tests were performed between 1 × 10^8^ and 10 × 10^8^ bacteria-infected larvae groups (**, *P < *0.01). (E) Representative images of guts from noninfected larvae and larvae infected with mCherry-*S aureus* USA300 WT (carrying pRN11 plasmid) for 6 h (6 h p.i.). Animals were dissected and stained with Alexa Fluor 488 phalloidin (green) and DAPI (blue) (*n* = 2, 10 guts/experiment, for each condition). Scale bar, 0.5 mm. AM, anterior midgut; MM, middle midgut; PM, posterior midgut. (F) Representative Lightsheet microscope images (20×/NA 0.1 objective) from the posterior part (ventral view) of a larva infected with mCherry-S. aureus USA300 WT at 6 h p.i. F1, F2, and F3 correspond, respectively, to a frontal plane (ventral view), transversal planes (reflecting the line’s disposition in F1), and the sagittal plane (extended view). Scale bar, 100 μm. (The experiment was performed on 5 animals on 5 different inclusions).

We established a 24-h infection course (Fig. 1A), after a 30-min period in which mid-L3 larvae were fed a mixture of mashed banana and bacteria (see Materials and Methods). We observed that after 24 h of infection, 93% of the larvae were killed by a bacterium-enriched medium containing 10 × 10^8^ bacteria, while lower bacterial doses (5 × 10^8^, 2.5 × 10^8^, or 1 × 10^8^ bacteria) killed only 62, 51, and 20% of the larvae, respectively (Fig. 1B). We then monitored the kinetics of larval killing using wild-type S. aureus USA300 (USA300 WT), compared with the Gram-positive opportunistic entomopathogen Micrococcus luteus, which is known to be nonpathogenic to D. melanogaster (47). Larvae were infected with medium enriched with 10 × 10^8^ bacteria for 30 min, and killing was followed over a 24-h period. Under these conditions, S. aureus USA300 WT was able to kill larvae, with a drop in animal survival occurring between 12 h and 18 h (Fig. 1C). In contrast, infection with M. luteus did not affect animal survival. We hypothesized that the death of the animals is related to the bacterial load in the gut. To avoid quantifying intestinal microbiota, we generated an S. aureus USA300 WT strain carrying the pRN11 plasmid, which expresses a chloramphenicol resistance (Cm^r^) gene (48). In support of the mortality data, the numbers of bacteria (CFU) in the larval gut were found to be 10-fold lower at 6 h and 20-fold lower at 24 h with an initial infective dose of 1 × 10^8^ bacteria than with an initial infective dose of 10 × 10^8^ bacteria (Fig. 1D). These results suggest that larval death is related to the number of bacteria in the gut. We then confirmed the absence of effective tracheal colonization. As shown in Fig. S1A in the supplemental material, numbers of CFU remained low in the tracheal system throughout the experiment, reaching the highest count at 6 h, with an average of 447 CFU for an infecting dose of 10 × 10^8^ bacteria. Furthermore, we showed that bacteria were not able to diffuse in the systemic compartment. As shown in Fig. S1B, S. aureus USA300 WT was almost undetectable in the hemolymph, as it reached only 8 and 6 CFU for 10 larvae, respectively, at 6 h and 18 h. Similar values were obtained with the nonpathogenic strain M. luteus (Fig. S1B). All together, these data indicate that S. aureus USA300 WT, in this model of oral infection, is pathogenic to D. melanogaster larva in a dose-dependent manner and that infection is constrained to the gut, where it persists for at least 24 h.

10.1128/mBio.00276-21.1 FIG S1 Infection is localized in the intestinal tract. (A) *w^1118^* D. melanogaster larvae were orally infected for 30 min with 1 × 10^8^ or 10 × 10^8^ chloramphenicol-resistant *S aureus* USA300 WT bacteria (carrying the pRN11 plasmid). Bacterial counts (CFU) in the trachea were determined at 6, 12, 18, and 24 h p.i. Tissues were homogenized in DPBS, serially diluted, and plated on BHI agar supplemented with chloramphenicol (10 μg · ml^−1^). Data are means ± SEM (*n* = 4). One-way ANOVA and then multiple-comparison tests were performed between larval groups infected with 1 × 10^8^ and 10 × 10^8^ bacteria (NS, not significant; *, *P < *0.05; **, *P < *0.01). (B) *w^1118^* D. melanogaster larvae were orally infected for 30 min with 10 × 10^8^ S. aureus USA300 WT bacteria/larva. Bacterial counts (CFU) in the trachea were determined at 6 and 18 h p.i. After an ethanol washing, animals were bled into DPBS, and hemolymph was plated on BHI agar. Data are means ± SEM (*n* = 3). One-way ANOVA and then multiple-comparison tests were performed between noninfected (NI) and infected groups. Download FIG S1, TIF file, 0.1 MB. Copyright © 2021 Ramond et al. 2021 Ramond et al. https://creativecommons.org/licenses/by/4.0/ This content is distributed under the terms of the Creative Commons Attribution 4.0 International license.

Since oral infection of adult flies with different S. aureus strains, including S. aureus USA300, does not interfere with animal survival (41, 42, 49), we assumed that larval death was related to a high number of ingested bacteria, due to their hyperphagic behavior. To confirm this, adult flies were first starved for 2 h and then placed on filters soaked with 10 × 10^8^ bacteria for 1 h. Neither S. aureus USA300 WT nor M. luteus oral infection affected the survival of adult flies (Fig. S2A). Indeed, after 1 h of feeding, numbers of CFU recorded were only about 2% of those recorded with larvae (i.e., 6.9 × 10^5^ bacteria/10 adult flies compared to 3.4 × 10^7^ bacteria/10 larvae) (Fig. S2B), suggesting that animal killing, when flies are orally infected by S. aureus USA300 WT, may depend on the bacterial load ingested. The number of bacteria counted at day 1 in adult flies is consistent with the results of the study from Hori et al. (42), where the authors retrieved 8 × 10^4^ bacteria per fly gut (compared to an average of 1.8 × 10^5^ bacteria per fly in our study). Of note, we found that at day 1, adult flies had bacteria in the middle midgut (Fig. S2C).

10.1128/mBio.00276-21.2 FIG S2 S. aureus USA300 is not pathogenic to adult Drosophila melanogaster flies. (A) Survival of *w^1118^* adult D. melanogaster flies after 1 h of feeding on an infectious dose of 10 × 10^8^ *S aureus* USA300 WT bacteria/larva or nonpathogenic strain M. luteus bacteria. Animals were monitored every day. Sixty-eight animals were pooled from 3 independent experiments. The Kaplan-Meier test was performed for the whole group. (B) *w^1118^* adult flies (5 to 7 days old) were orally infected for 1 h with 10 × 10^8^ chloramphenicol-resistant wild-type *S aureus* USA300 bacteria/larva. At the indicated time points, guts from 10 flies were homogenized and plated to enumerate intestinal bacteria. Day 0 corresponds to the end of the proper infection. Data are means ± SEM (*n* = 4). One-way ANOVA and then multiple-comparison tests were performed between days 1 and 8 versus day 0 (**, *P < *0.01; ***, *P < *0.001). (C) Representative confocal microscopy imaging of an adult fly intestine, 1 day after infection with mCherry-*S aureus* USA300 WT. Green, Alexa Fluor 488 phalloidin; red, bacteria expressing the mCherry protein. Scale bar = 0.5 mm. We used 15 intestines from 3 and 5 intestines per experiment. Download FIG S2, TIF file, 0.3 MB. Copyright © 2021 Ramond et al. 2021 Ramond et al. https://creativecommons.org/licenses/by/4.0/ This content is distributed under the terms of the Creative Commons Attribution 4.0 International license.

We next analyzed S. aureus localization in the larval gut using fluorescence microscopy (Fig. 1E) and Lightsheet three-dimensional (3D) imaging (Fig. 1F and Movie S1). For this, we used the S. aureus USA300 WT strain carrying the pRN11 plasmid, which expresses the *mCherry* gene (48) (red fluorescence). Imaging from 6-h-infected larvae with *mCherry*-expressing S. aureus USA300 WT revealed that bacteria were clustered in the posterior midgut (Fig. 1E and F and Movie S1). This specific localization in larvae might be explained by the local pHs of the gut, as the first half of the hind midgut is at neutral to acidic pH, compared with the middle midgut, which corresponds to a very acidic region (pH < 3), and the second half of the posterior midgut, which corresponds to an alkaline region (pH > 10) (50). To test this hypothesis, we tested S. aureus USA300 susceptibility to a wide range of different pHs (from 3 to 11) (Fig. S3). We observed that S. aureus USA300 was highly susceptible to the highly acidic pH 3, as well as to basic pH 11. In contrast, bacteria were able to survive at pH 5 and 9 for at least 2 h and multiplied at pH 7. This sensitivity to environmental pH may explain the specific localization of S. aureus USA300 WT in the neutral region of the *Drosophila* larval gut. Interestingly, the site of infection was associated with apparent inflammation (a swelling of the gut in this area of about 1.3 times that of uninfected larvae) (Fig. S4). Overall, these data show that S. aureus USA300 successfully colonizes D. melanogaster larvae after a 24-h infection and preferentially localizes to the anterior half of the midgut. This prolonged infection results in tissue inflammation and correlates with animal death.

10.1128/mBio.00276-21.3 FIG S3 pH tolerance assay of S. aureus USA300. Bacteria were inoculated at 2 × 10^7^ bacteria/ml in BHI broth, the pH of which was adjusted to 3, 5, 7, 9, or 11. Susceptibility was checked at 30, 60, 90, and 120 min postinoculation. Data are means ± SEM (*n* = 3). One-way ANOVA and then multiple-comparison tests were performed between the pH 7 group and the pH 3, 5, 9, or 11 group for each time point (*, *P < *0.05; ***, *P < *0.001). Download FIG S3, TIF file, 0.1 MB. Copyright © 2021 Ramond et al. 2021 Ramond et al. https://creativecommons.org/licenses/by/4.0/ This content is distributed under the terms of the Creative Commons Attribution 4.0 International license.

10.1128/mBio.00276-21.4 FIG S4 Intestinal inflammation following S. aureus USA300 infection. The intestinal thickness of the posterior midgut from noninfected animals was measured 6 h p.i. with S. aureus USA300 WT. Each point corresponds to one animal. Six animals were dissected for each condition in 3 independent experiments; data were pooled (*n* = 18). Data are means ± SEM (*n* = 3). The Mann-Whitney test was performed between WT-infected and noninfected larvae groups (***, *P < *0.001). Download FIG S4, TIF file, 0.05 MB. Copyright © 2021 Ramond et al. 2021 Ramond et al. https://creativecommons.org/licenses/by/4.0/ This content is distributed under the terms of the Creative Commons Attribution 4.0 International license.

10.1128/mBio.00276-21.8 MOVIE S1 Related to Fig. 1E. Lightsheet-based imaging of a larva infected with S. aureus. Representative posterior mid-half visualization (ventral view) of a mid-L3 larva infected with *mCherry*-expressing S. aureus USA300 WT at the infectious dose of 10 × 10^8^ bacteria for 30 min. At 6 h p.i., animals where fixed and cleared for further Lightsheet imaging. Bacteria localize specifically in the intestinal posterior part of the animal. Download Movie S1, MOV file, 3.8 MB. Copyright © 2021 Ramond et al. 2021 Ramond et al. https://creativecommons.org/licenses/by/4.0/ This content is distributed under the terms of the Creative Commons Attribution 4.0 International license.

### *Drosophila* larvae are a suitable model for human pathogen studies.

To assess the versatility of the larval model and confirm its ease of implementation, we tested the infection of larvae with three other clinical S. aureus strains belonging to different sequence types (STs) that are predominant among clinical S. aureus isolates sampled in human infections. These strains have been designated as follows: S. aureus P1 (ST1), S. aureus P2 (ST5), and S. aureus P3 (ST30) (see Materials and Methods for details). As shown in Fig. S5A, we observed that animals infected with S. aureus P1 and P2 followed survival curves identical to those of larvae infected with S. aureus USA300 WT (with, respectively, 24, 23, and 31% of animals surviving at 24 h postinfection [p.i.]), while S. aureus P3 showed a slightly higher virulence (with 9% of larvae surviving at 24 h p.i.). No difference in bacterial survival was observed (the CFU counts recorded for the three isolates at 6 h and 24 h p.i. were identical to those of the WT strain) (Fig. S5B). Interestingly, the three isolates differentially activated the *Drosophila* Toll pathway. At 6 h p.i., both S. aureus P1 and P2 triggered a 44% decrease in the expression of the antimicrobial peptide drosomycin (*Drs*; one of the main readouts for the Toll pathway in D. melanogaster) compared with that of S. aureus USA300 WT, whereas S. aureus P3 induced a 66% increase in the expression of *Drs* (Fig. S5C). These data suggest that the *Drosophila* larvae can serve as a model to evaluate the virulence of S. aureus clinical isolates as well as their ability to activate the host immune response.

10.1128/mBio.00276-21.5 FIG S5 D. melanogaster larva as a model organism to study S. aureus clinical isolates. (A) Survival of *w^1118^* D. melanogaster larvae upon 30 min of oral infection with 10 × 10^8^ S. aureus USA300 WT or S. aureus P1, P2, or P3 bacteria. Animals were monitored at 0, 6, 12, 18, and 24 h after infection. At least 60 animals from 3 independent experiments were pooled. The Kaplan-Meier test was applied to the whole group (***, *P < *0.001). (B) *w^1118^* D. melanogaster larvae were orally infected for 30 min with 10 × 10^8^ S. aureus USA300 WT or S. aureus P1, P2, or P3 bacteria/larva. Bacterial counts (CFU) in the gut were determined at 6 and 24 h p.i. Tissues were homogenized in DPBS, serially diluted, and plated on supplemented chromID MRSA agar. Data are means ± SEM. (C) *w^1118^* mid-L3 larvae were fed for 30 min with bacteria at the infectious dose of 10 × 10^8^ bacteria/larva. Quantitative real-time PCR analysis on *Drosomycin* transcripts was done with total RNA extracts from guts recovered at 6 h p.i. Bar graph data are presented related to *RP49*. Data are means ± SEM (*n* = 3). One-way ANOVA and then multiple-comparison tests were performed between infected groups and the noninfected group (*, *P < *0.05; ***, *P < *0.001). Download FIG S5, TIF file, 0.1 MB. Copyright © 2021 Ramond et al. 2021 Ramond et al. https://creativecommons.org/licenses/by/4.0/ This content is distributed under the terms of the Creative Commons Attribution 4.0 International license.

We have also tested the value of this model with two human enteric pathogens: Salmonella enterica serovar Typhimurium and Shigella flexneri. Notably we observed significant larval death when organisms were fed 10 × 10^8^ bacteria/larva, under conditions similar to those of S. aureus infection (see Materials and Methods). Indeed, after 24 h of infection, 53.8% and 46.6% of the larvae were killed, respectively, when they were fed *S.* Typhimurium- and S. flexneri*-*enriched medium (Fig. S6A). After 30 min of feeding, the larvae were infected with 8.2 × 10^6^ *S.* Typhimurium or 7.9 × 10^6^ S. flexneri bacteria per 10 larvae. At 6 h p.i., 5 × 10^5^ and 2 × 10^5^ bacteria per 10 larvae were recorded, respectively (Fig. S6B). We confirmed that these two pathogens also triggered immune responses in *Drosophila*, as we observed a significant production of intestinal H_2_O_2_ at 2 h when larvae were infected with *S.* Typhimurium (43% increase) and S. flexneri (57% increase) (Fig. S6C). This correlated with a significant increase in the expression of the gene for the antimicrobial peptide diptericin (*Dpt*), which is dependent on the Gram-negative, sensitive immune deficiency pathway (51). We observed 42.9-fold (*S.* Typhimurium) and 37.9-fold (S. flexneri) increases in *Dpt* expression at 6 h postinfection (Fig. S6D). Interestingly, by using DsRed-expressing strains, we observed that at 6 h postinfection, each strain preferentially localized to the foregut and midgut (Fig. S6E, white arrowheads), where pH values ranged from neutral to acidic (50). This trait may be explained by their ability to survive in an acidic environment (52). We confirmed that the sensitivity of *S*. Typhimurium and S. flexneri to pH 5 after 2 h of treatment (Fig. S6F) was lower than that of S. aureus. After 2 h in brain heart infusion (BHI) broth adjusted to pH 5, the number of CFU of S. aureus was 25 times lower than after treatment at pH 7. In contrast, for *S*. Typhimurium and S. flexneri, the values recorded at pH 5 were only 5- to 3-fold lower than those recorded at pH 7, respectively.

10.1128/mBio.00276-21.6 FIG S6 D. melanogaster larva can serve as a model host for human enteric pathogens. (A) Survival of *w^1118^* D. melanogaster larvae upon 30 min of oral infection with 10 × 10^8^ S. aureus USA300, *S.* Typhimurium, or S. flexneri bacteria. Animals were monitored at 0, 6, 12, 18, and 24 h after infection. Sixty-five to 70 animals from 3 independent experiments were pooled. The Kaplan-Meier test was applied to the whole group (***, *P < *0.001). (B) *w^1118^* D. melanogaster larvae were orally infected for 30 min with 10 × 10^8^ chloramphenicol-resistant S. aureus USA300 (carrying the pRN11 plasmid) or ampicillin-resistant *S.* Typhimurium and S. flexneri bacteria. Bacterial counts (CFU) in the gut were determined at 0.5 and 6 h p.i. Tissues were homogenized in DPBS, serially diluted, and plated on BHI agar supplemented with chloramphenicol (10 μg · ml^−1^) or ampicillin (100 μg · ml^−1^). Data are means ± SEM (*n* = 3). One-way ANOVA and then multiple-comparison tests were performed between infected animals recovered at 0.5 h and 6 h (**, *P < *0.01). (C) *w^1118^* mid-L3 larvae were fed for 30 min with 10 × 10^8^ *S.* Typhimurium or S. flexneri bacteria. Generation of intestinal H_2_O_2_ was measured with the H_2_DCFDA dye (10 μM) in noninfected samples at 2 and 6 h p.i. Data are means ± SEM (*n* = 3). One-way ANOVA and then multiple-comparison tests were performed between groups infected with the same bacteria and the respective noninfected group (*, *P < *0.05; **, *P < *0.01). (D) *w^1118^* mid-L3 larvae were fed for 30 min with bacteria at the infectious dose of 10 × 10^8^ bacteria. Quantitative real-time PCR analysis of *Diptericin* transcripts was done with total RNA extracts from guts recovered at 6 h p.i. Bar graph data are presented relative to *RP49*. Data are means ± SEM (*n* = 4). One-way ANOVA and then multiple-comparison tests were performed between infected groups and the noninfected group (**, *P < *0.01). (E) Representative images of guts from 6-h-infected larvae with DsRed *S.* Typhimurium or S. flexneri. Animals were dissected and stained with Alexa Fluor 488 phalloidin (green) and DAPI (blue) (*n* = 3, with 25 guts in total for each condition). Scale bar = 0.5 mm. (F) *S.* Typhimurium (left) and S. flexneri (right) were inoculated at 2 × 10^7^ bacteria/ml in BHI broth, the pH of which was adjusted to 3, 5, 7, 9, or 11. Susceptibility was checked at 30, 60, 90, and 120 min postinoculation. (E and F) Data are means ± SEM (*n* = 3). Two-way ANOVA and then multiple-comparison tests were performed between the groups treated with medium at pH 3, 5, 9, or 11 and the group treated with medium at pH 7 for each time point (**, *P < *0.01; ***, *P < *0.001). Download FIG S6, TIF file, 11.7 MB. Copyright © 2021 Ramond et al. 2021 Ramond et al. https://creativecommons.org/licenses/by/4.0/ This content is distributed under the terms of the Creative Commons Attribution 4.0 International license.

### S. aureus USA300 triggers host intestinal hydrogen peroxide production.

**FIG 2:**
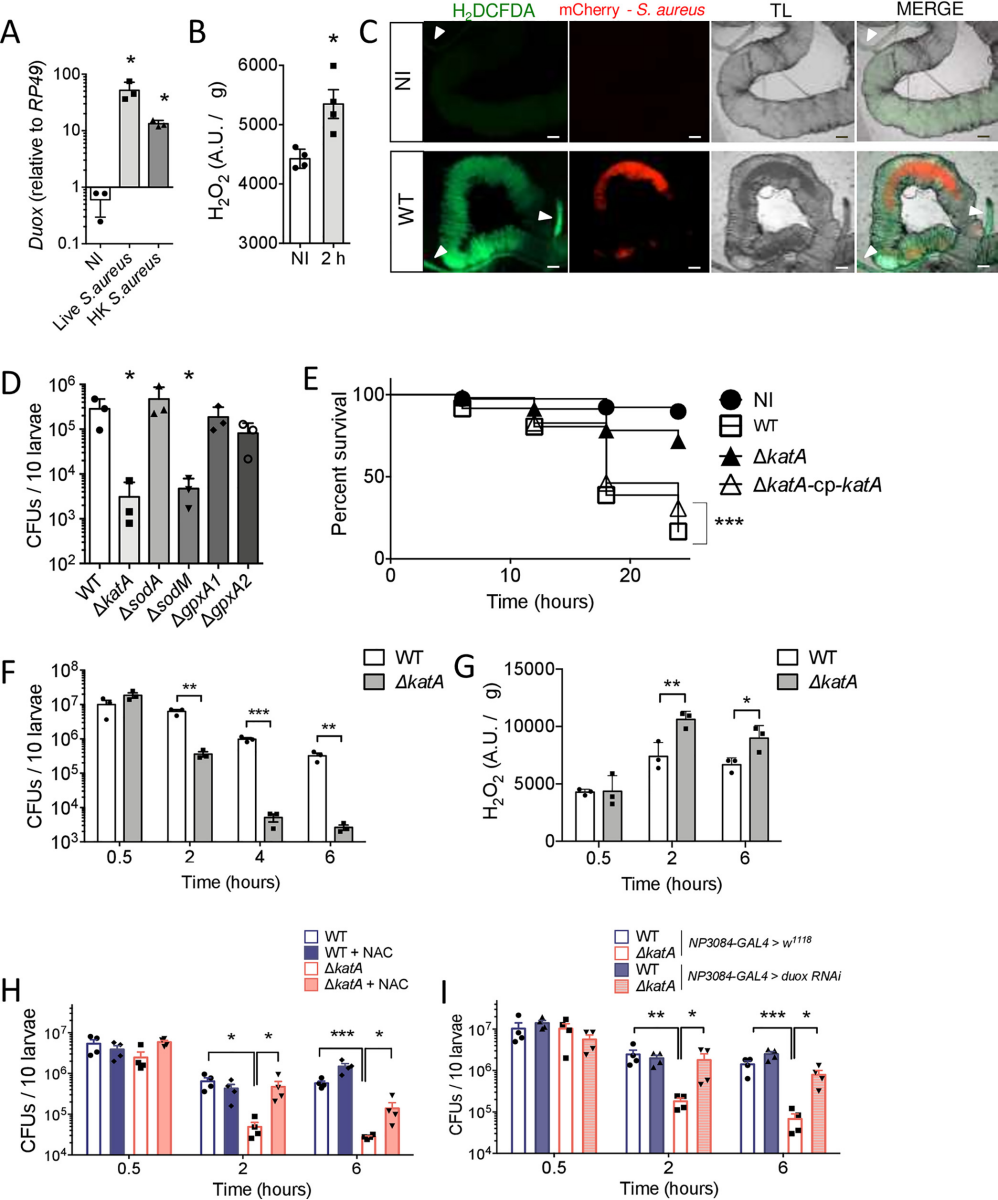
ROS quenching *in vivo* is a key mechanism for successful colonization. (A) *w^1118^* mid-L3 larvae were fed for 30 min with 10 × 10^8^ live or heat-killed (HK) S. aureus USA300 WT bacteria/larva. A quantitative real-time PCR analysis of *Duox* transcripts was done with total RNA extracts from guts (15 animals, *n* = 3) recovered at 2 h p.i. Bar graph data are relative to *RP49*. Data are means ± SEM (*n* = 3). One-way ANOVA and then multiple-comparison tests between infected groups and noninfected (NI) larvae were applied (*, *P < *0.05; **, *P < *0.01). (B) *w^1118^* mid-L3 larvae were fed for 30 min with 10 × 10^8^ *S aureus* USA300 WT bacteria/larva. Generation of intestinal H_2_O_2_ was measured with the H_2_DCFDA dye (10 μM) on noninfected samples and at 2 h p.i. Data are means ± SEM (*n* = 4). The Mann-Whitney test was applied (*, *P < *0.05). A.U., arbitrary units. (C) Representative live imaging of posterior midguts from noninfected larvae (NI) and orally infected larvae (2 h p.i.; mCherry-S. aureus USA300 WT, red). Intestines were dissected, treated with H_2_DCFDA (10 μM, green) for 15 min, and imaged with an epifluorescence microscope. TL, transmitted light. White arrowheads indicate Malpighian tubules. Scale bar, 10 μm. (*n* = 3, 10 guts/experiment, for each condition.) (D) *w^1118^* D. melanogaster larvae were orally infected for 30 min at 10 × 10^8^ bacteria/larva with chloramphenicol-resistant *S aureus* USA300 WT or the Δ*katA*, Δ*sodA*, Δ*sodM*, Δ*gpxA1*, or Δ*gpxA2* (carrying the pRN11 plasmid) strain. Bacterial counts (CFU) were determined at 0 6 h p.i. After homogenization and serial dilution, samples were plated on BHI agar supplemented with chloramphenicol (10 μg · ml^−1^). Data are means ± SEM (*n* = 3). One-way ANOVA and then multiple-comparison tests between mutant-infected groups and the WT-infected group were applied (*, *P < *0.05). (E) Survival of *w^1118^* D. melanogaster larvae following 30 min of oral infection with S. aureus USA300 WT Δ*katA* Δ*katA*-cp-*katA* with 10 × 10^8^ bacteria/larva. The experiment was monitored until 24 h after infection. Sixty-two animals were pooled from 3 independent experiments. The Kaplan-Meier test was applied for the whole group (***, *P < *0.001). (F) *w^1118^* D. melanogaster larvae were orally infected for 30 min at 10 × 10^8^ bacteria/larva with chloramphenicol-resistant *S aureus* USA300 WT or the Δ*katA* mutant (carrying the pRN11 plasmid). Bacterial counts (CFU) were determined at 0.5, 2, 4, and 6 h p.i. After homogenization and serial dilution, samples were plated on BHI agar supplemented with chloramphenicol (10 μg · ml^−1^). Data are means ± SEM (*n* = 3). One-way ANOVA and then multiple-comparison tests were performed between WT and Δ*katA*-infected larval groups (**, *P < *0.01; ***, *P < *0.001). (G) *w^1118^* mid-L3 larvae were fed for 30 min with chloramphenicol-resistant S. aureus USA300 WT or the Δ*katA* mutant at the infectious dose of 10 × 10^8^ bacteria/larva. The intestinal ROS titer was measured at 0.5, 2, and 6 h p.i. After dissection, intestines were homogenized in 400 μl DPBS and treated with H_2_DCFDA (10 μM) for 30 min. Fluorescence was measured at 490 nm. Data are means ± SEM (*n* = 3). One-way ANOVA and then multiple-comparison tests were performed between WT- and Δ*katA*-infected larval groups (*, *P < *0.05; **, *P < *0.01). (H) *w^1118^* D. melanogaster mid-L3 larvae were orally infected for 30 min with chloramphenicol-resistant S. aureus USA300 WT or the Δ*katA* mutant (carrying the pRN11 plasmid) at the infectious dose of 10 × 10^8^ bacteria/larva. Then animals were transferred to fresh fly medium supplemented, or not, with NAC (1 mM). Bacterial counts (CFU) were determined at 0.5, 2, and 6 h p.i. After homogenization and serial dilution, samples were plated on BHI agar supplemented with chloramphenicol (10 μg · ml^−1^). Data are means ± SEM (*n* = 4). One-way ANOVA and then multiple-comparison tests were performed (*, *P < *0.05; ***, *P < *0.001). (I) *NP3084-GAL4 *>* w^1118^* and *NP3084-GAL4 *>* Duox RNAi* larvae were orally infected for 30 min with chloramphenicol-resistant S. aureus USA300 WT or the Δ*katA* mutant (carrying the pRN11 plasmid) at the infectious dose of 10 × 10^8^ bacteria/larva. Bacterial counts (CFU) were determined at 0.5, 2, and 6 h p.i. After homogenization and serial dilution, samples were plated on BHI agar supplemented with chloramphenicol (10 μg · ml^−1^). Data are means ± SEM (*n* = 4). One-way ANOVA and then multiple-comparison tests were performed (*, *P < *0.05; **, *P < *0.01; ***, *P < *0.001).

It has previously been shown that adult intestinal infection triggers the production of reactive oxygen species (ROS) by the Duox enzyme to eliminate invading pathogens, complementing AMP actions (53). Therefore, we monitored the transcripts level of the *Duox* gene in intestines from infected larvae (10 × 10^8^ bacteria). As shown in Fig. 2A, we observed an 86.8-fold increase in *Duox* transcription at 2 h p.i. over that of noninfected animals. Interestingly, the use of heat-killed (HK) bacteria also induced *Duox* transcription but to a lesser extent (22.2-fold increase), suggesting that the S. aureus cell wall, as well as secreted factors, modulates *Duox* transcription. To confirm this induction of intestinal ROS in this context, we used the H_2_O_2_-specific detector 2′,7′-dichlorodihydrofluorescein diacetate (H_2_DCFDA). We observed a 20% increase in signal detection in infected intestines 2 h p.i. compared to that in noninfected intestines (Fig. 2B). This was confirmed by live imaging (Fig. 2C). Interestingly, H_2_DCFDA fluorescence (green) often colocalized with bacteria (mCherry S. aureus USA300, red) in the intestine. We also noticed strong H_2_DCFDA fluorescence in Malpighian tubules when animals were infected (Fig. 2C, white arrowheads). Of note, it was recently shown that Malphigian tubules also play an active role during oral infection by sequestering excessive ROS and oxidized lipids (54). All together, these results demonstrate that S. aureus USA300 oral infection rapidly triggers H_2_O_2_ production at the intestinal epithelium, through Duox activation.

### Catalase is a key enzyme in the S. aureus antioxidant defense.

Since H_2_O_2_ generation through Duox enzyme is a key mechanism for controlling pathogen load (55), we then tested which S. aureus antioxidant enzymes, catalase (*katA* encoded), superoxide dismutases (*sodA* and *sodM* encoded), or glutathione peroxidases (*gpxA1* and *gpxA2* encoded), might contribute to intestinal persistence during host infection. For this, larvae were orally infected with *katA*, *sodA*, *sodM*, *gpxA1*, or *gpxA2* transposon insertion mutants (kindly obtained from BEI Resources [see Materials and Methods]), and we evaluated bacterial persistence at 6 h p.i. We used pRN11-transformed bacteria to allow chloramphenicol resistance gene expression and specific clone selection on chloramphenicol-supplemented BHI agar.

The Δ*sodA*, Δ*gpxA1*, and Δ*gpxA2* mutants behaved like the wild-type strain. In contrast, the growth of the Δ*sodM* mutant was 60-fold less than that of the WT strain (Fig. 2D), supporting an earlier report of a mouse abscess model (17). The Δ*katA* mutant was the most attenuated strain compared to the WT strain (92-fold), prompting us to evaluate its role in S. aureus virulence and persistence. Genome-wide sequencing and analysis of the Δ*katA* mutant confirmed the transposon insertion site in the *katA* gene and the absence of unintended secondary mutations. We showed that the Δ*katA* mutant strain was more sensitive to H_2_O_2_ than the WT strain (15 mM H_2_O_2_ in Dulbecco’s phosphate-buffered saline [DPBS]) (Fig. S7A). As shown in Fig. 2E, we observed that S. aureus USA300 Δ*katA* killed larvae to a much lesser extent than the WT strain. However, expression of the catalase gene in the mutant strain restored its virulence phenotype. This difference in larval survival was correlated with 17.5-, 190-, and 122-fold decreases in the number of intestinal Δ*katA* mutant CFU compared to the number of WT CFU, respectively, at 2, 4, and 6 h p.i. (Fig. 2F). Restoration of catalase expression in the Δ*katA* strain restored S. aureus survival in the *Drosophila* gut to WT S. aureus levels at 6 h p.i. (Fig. S7B). Supporting the idea that this greater bacterial clearance may be related to a defect in H_2_O_2_ quenching, we observed a significant increase in the amount of ROS, by H_2_DCFDA measurement, in the intestines of Δ*katA* mutant-infected larvae compared to that in WT-infected larvae (with respective increases in fluorescence intensity of 43% and 34% at 2 and 6 h p.i.) (Fig. 2G). These results suggest that S. aureus USA300 Δ*katA* is defective for H_2_O_2_ quenching. Second, we confirmed that bacterial persistence in the larval gut and the bacterium’s ability to kill larvae are closely related to ROS content. For this, we evaluated numbers of bacterial CFU of the WT and Δ*katA* strains in flies fed *N*-acetyl-l-cysteine (NAC; 1 mM), an antioxidant drug that was shown to quench H_2_O_2_ molecules (56). We observed that NAC counteracted the deleterious intestinal environment for the Δ*katA* strain, as NAC abolished the Δ*katA* mutant’s defect compared to the WT at 2 h p.i. and promoted a 5-fold increase in the CFU count of the Δ*katA* mutant at 6 h p.i. (Fig. 2H). In parallel, we tested the survival of the WT and Δ*katA* strain in *NP3084-GAL4 > Duox-RNAi* larvae that are defective for *Duox* expression specifically in the intestine. *NP3084-GAL4* (or *MyoD1-GAL4*) primarily drives gene expression in the larval midgut in enterocytes (57). Notably, larvae with abolished Duox expression in the midgut (*NP3084-GAL4 > Duox-RNAi*) showed significant 9.8- and 11.7-fold increases in CFU counts for the Δ*katA* strain, respectively, at 2 h and 6 h p.i. in *NP3084-GAL4 > Duox-RNAi* compared to counts in *NP3084-GAL4 > w^1118^* larvae. In contrast, the S. aureus USA300 WT strain showed nonsignificant 0.8- and 1.7-fold changes in CFU counts in *NP308-GAL4 > Duox-RNAi* larvae from those of *NP3084-GAL4 > w^1118^* larvae (Fig. 2I). Taken together, these results indicate that oral infection induces H_2_O_2_ generation from the epithelial barrier, which acts as a key mechanism to control the growth of the pathogen. Furthermore, catalase activity is paramount for S. aureus resistance to the host response by H_2_O_2_ quenching.

10.1128/mBio.00276-21.7 FIG S7 S. aureus USA300 Δ*katA* characterization. (A) Exponential-phase bacteria were diluted at the concentration 2 × 10^7^ bacteria/ml and tested for H_2_O_2_ survival in DPBS (15 mM). Bacteria were plated for 30 min on BHI agar. Data are means ± SEM (*n* = 3). Two-way ANOVA and then multiple-comparison tests were performed as indicated (***, *P < *0). (B) *w^1118^* D. melanogaster larvae were orally infected for 30 min with 10 × 10^8^ *S aureus* USA300 WT (carrying the pRN11 plasmid), Δ*katA*, or Δ*katA-*cp-*katA* bacteria/larva. Bacterial counts (CFU) in the trachea were determined at 6 h p.i. They were plated on chloramphenicol (WT)- or erythromycin (Δ*katA* and Δ*katA-*cp-*katA*)-supplemented BHI. Data are means ± SEM (*n* = 3). One-way ANOVA and then multiple-comparison tests were performed between the WT or Δ*katA-*cp-*katA* strain and the Δ*katA* strain (*, *P < *0.05). Download FIG S7, TIF file, 0.1 MB. Copyright © 2021 Ramond et al. 2021 Ramond et al. https://creativecommons.org/licenses/by/4.0/ This content is distributed under the terms of the Creative Commons Attribution 4.0 International license.

### Catalase-mediated ROS quenching limits Toll pathway activation in the host.

**FIG 3:**
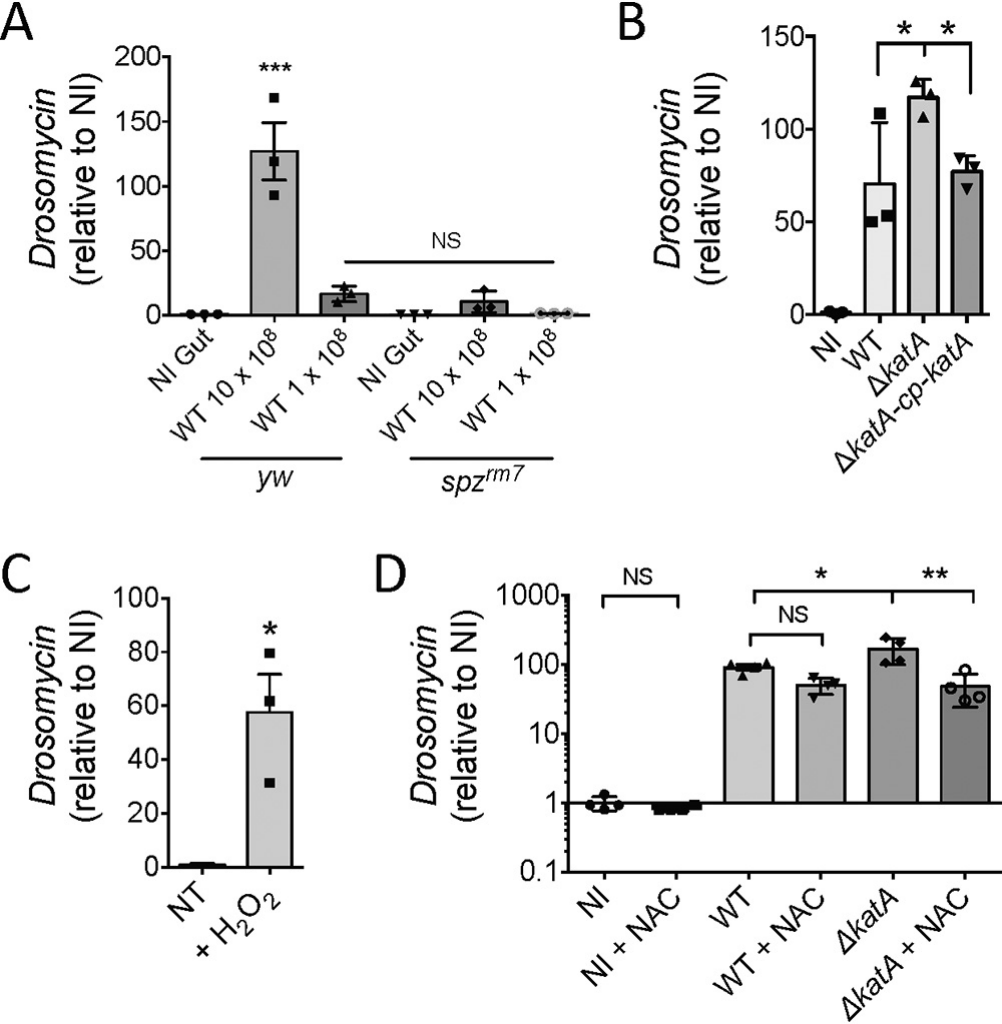
S. aureus USA300 modulates the antimicrobial response by neutralizing intestinal ROS. (A) *yw* and *yw*;;*spz^rm7^* mid-L3 larvae were fed for 30 min with the infectious doses of 1 × 10^8^ and 10 × 10^8^ bacteria/larva. Quantitative real-time PCR analysis of *Drosomycin* transcripts was done with total RNA extracts from guts recovered at 6 h p.i. Bar graph data are presented in relation to *RP49*. Data are means ± SEM (*n* = 3). One-way ANOVA and then multiple-comparison tests were performed between the “*yw* NI Gut” condition” and all other conditions (NS, nonsignificant; ***, *P < *0.001). (B) *w^1118^* mid-L3 larvae were orally infected for 30 min with *S aureus* USA300 WT or the Δ*katA* or Δ*katA-*cp-*katA* strain at the infectious dose of 10 × 10^8^ bacteria/larva. At 6 h p.i., guts were dissected for quantitative real-time PCR analysis of *Drosomycin* transcripts. Data were normalized to the corresponding *RP49* levels. Data are means ± SEM (*n* = 3). One-way ANOVA and then multiple-comparison tests were performed (*, *P < *0.05). (C) *w^1118^* mid-L3 larvae were fed for 2 h with fly medium supplemented with stabilized H_2_O_2_ (0.5%). Guts were dissected for quantitative real-time PCR analysis of *Drosomycin* transcripts. Transcripts levels were normalized to the corresponding *RP49* levels. Data are means ± SEM (*n* = 3). The Mann-Whitney test was applied to compare the nontreated (NT) group and H_2_O_2_-treated group (*, *P < *0.05). (D) *w^1118^* mid-L3 larvae were orally infected for 30 min with *S aureus* USA300 WT or S. aureus USA300 Δ*katA* at the infectious dose of 10 × 10^8^ bacteria/larva. Then animals were transferred to fresh fly medium supplemented, or not, with NAC (1 mM). At 6 h p.i., guts were dissected for quantitative real-time PCR analysis of *Drosomycin* transcripts. Data were normalized to the corresponding *RP49* levels. Results were compared to those for noninfected larvae transferred to supplemented NAC medium (NI+NAC) or not (NI). Data are means ± SEM (*n* = 4). One-way ANOVA and then multiple-comparison tests were performed (*, *P < *0.05; **, *P < *0.01).

Like other Gram-positive bacteria, S. aureus is known to induce the Toll pathway, a key innate immune signaling pathway in D. melanogaster, through its lysine-type peptidoglycan (58). This prompted us to test the expression of the *Drs* gene in wild-type *yw* larvae and in the derivative *spz^rm7^* mutated line (larvae lacking the expression of the Toll ligand spätzle) when larvae were infected with the S. aureus USA300 WT strain. As shown in Fig. 3A, we observed a significant 126-fold increase in intestinal *Drs* expression, in comparison to levels under noninfected conditions (using a 10 × 10^8^ bacteria/larva setup), in *yw* flies. This activation was proportional to the initial bacterial load, as a 10-fold-lower infectious dose (1 × 10^8^) induced only a 16-fold increase in *Drs* gene transcription. Notably, using *spz^rm7^* larvae considerably reduced the *Drs* transcript amount, even using medium enriched with 10 × 10^8^ bacteria, suggesting that *Drs* activation is almost exclusively controlled by the Toll pathway. In *Drosophila*, links between ROS and the Toll/NF-κB pathway have already been established. Under wasp infestation (at the larval stage), the lymph gland (the main hematopoietic organ) undergoes a burst of ROS in the posterior signaling center, resulting in Toll pathway activation, whose purpose is to redirect hemocyte progenitor differentiation into the lamellocyte subtype (59). This led us to wonder if H_2_O_2_ generated during the infection plays a role in Toll pathway activation in the intestine. We first evaluated *Drs* expression in animals infected with S. aureus USA300 WT or the Δ*katA* or Δ*katA* cp *katA* strain (S. aureus USA300 Δ*katA* complemented with the pCN57-cp-*katA* plasmid [see Materials and Methods]). Interestingly, the Δ*katA* strain induced a 65% increase in *Drs* transcription at 6 h p.i. in *Drosophila* intestine compared to that in WT-infected animals. Infection with the complemented Δ*katA* strain allowed restoration of *Drs* expression to levels under WT-infected conditions (Fig. 3B). We then tested the direct effect of H_2_O_2_ on intestinal *Drs* expression. Animals treated with H_2_O_2_ (0.5% in fly medium) for 2 h showed a 15-fold induction of *Drs* expression in the gut (Fig. 3C). We then evaluated the expression of the *Drs* gene in flies infected with the WT and Δ*katA* strains, fed with NAC or not. At 6 h p.i., the WT and Δ*katA* strains induced 91- and 169-fold increases in *Drs* expression, respectively, relative to levels under noninfected condition. Of note, at this time point (Fig. 2F), the number of Δ*katA* bacteria was 122-fold lower than that of the WT strain, suggesting that factors other than the bacteria themselves modulate *Drs* expression. Feeding animals with NAC induced relative 1.8- and 3.5-fold decreases in *Drs* expression in larval intestines at 6 h p.i. when larvae were infected, respectively, with the Δ*katA* and WT strains (Fig. 3D).

### The *katA* gene is differentially regulated by SigB and Agr *in vitro* and *in vivo*.

We then tested the contribution of the master regulators SigB and Agr in the virulence of S. aureus in fly larvae and more particularly their influence on the expression of catalase encoded by *katA*. The expression of the alternative factor sigma B is linked to environmental stress and plays a key role in resistance to oxidative stress, heat, and antibiotics, while the two-component quorum-sensing system encoded by the accessory gene regulator (the Agr loci, composed of the AgrA, AgrB, AgrC, and AgrD loci) locus regulates multiple virulence components (60). To address their role, we used *sigB* and *agrC* (*agrC* codes for the receptor histidine kinase AgrC) transposon insertion mutants. As for the *katA* transposon insertion mutant, the whole-genome sequence analysis of the *agrC* and *sigB* transposon insertion mutants confirmed the correct transposon insertion site and the absence of unintended mutations in these mutant strains.

**FIG 4:**
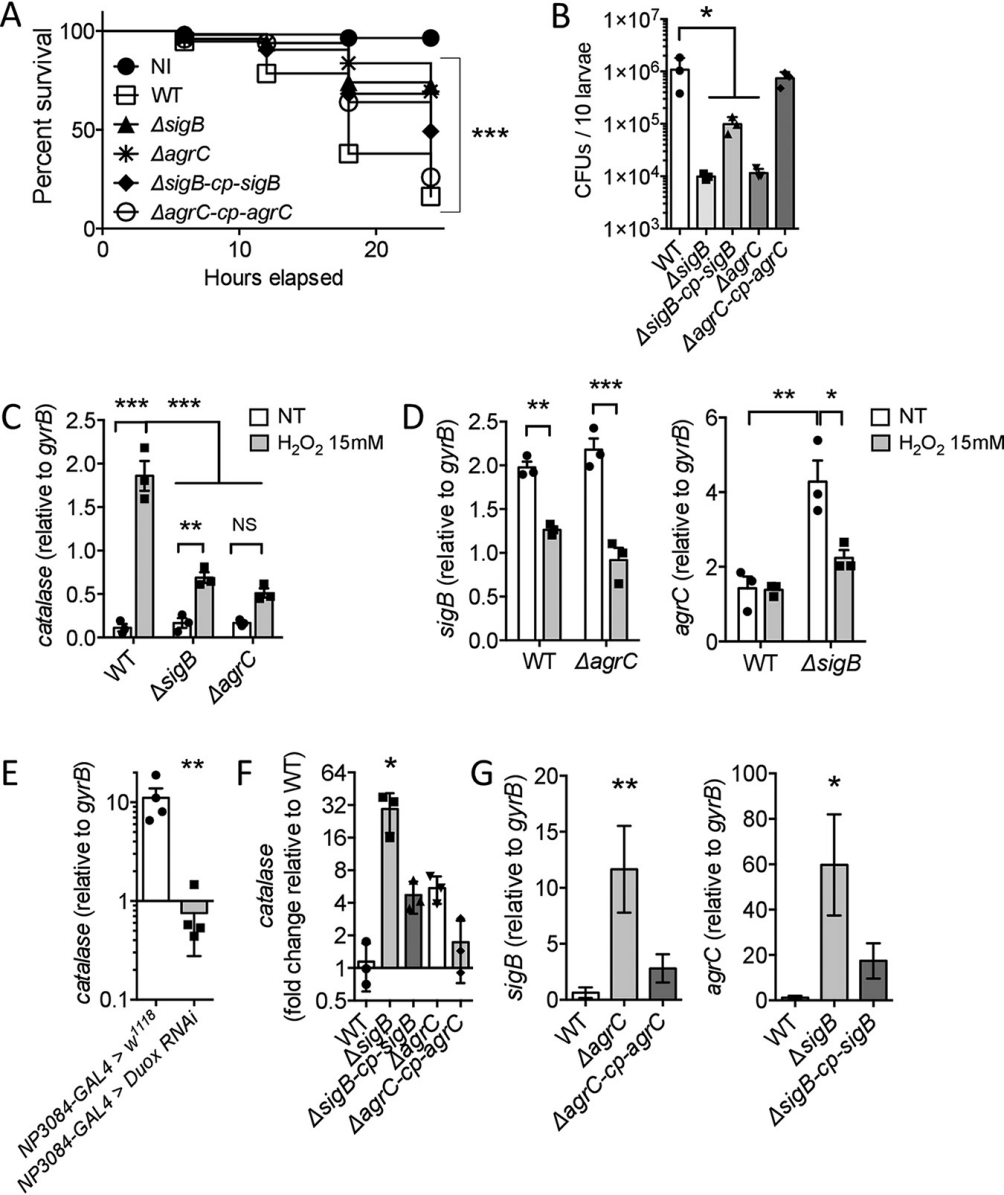
*katA* expression is differently mediated *in vitro* and *in vivo.* (A) Survival of *w^1118^* D. melanogaster larvae following 30 min of oral infection (10 × 10^8^ bacteria/larva) with S. aureus USA300 WT or the Δ*sigB*, Δ*sigB-cp-sigB*, Δ*agrC*, or Δ*agrC-cp-agrC* strain against noninfected animals (NI). The experiment was monitored until 24 h after infection. At least 60 animals were pooled under each condition from 3 independent experiments. The Kaplan-Meier test was used to compare the whole group (***, *P < *0.001). (B) *w^1118^* D. melanogaster larvae were orally infected for 30 min at 10 × 10^8^ bacteria/larva with chloramphenicol-resistant *S aureus* USA300 WT (carrying the pRN11 plasmid, plated on BHI agar with chloramphenicol) or the Δ*sigB*, Δ*sigB-cp-sigB*, Δ*agrC*, or Δ*agrC-cp-agrC* strain (plated on BHI agar with erythromycin). Bacterial counts (CFU) were determined at 6 h p.i. Data are means ± SEM (*n* = 3). One-way ANOVA and then multiple-comparison tests were performed (*, *P < *0.05). (C, D) Exponentially grown S. aureus WT (C), the Δ*sigB* mutant (D, left panel), or the Δ*agrC* mutant (D, right panel) were incubated for 30 min in DPBS with or without (NT) H_2_O_2_ 15 mM. Quantitative real-time PCR analyses of *katA* (C), *sigB* (D, left panel), or *agrC* (D, right panel) transcripts were performed. Transcripts levels were normalized to the corresponding *gyrB* levels. Data are means ± SEM (*n* = 3). One-way ANOVA and then multiple-comparison tests were performed (*, *P < *0.05; **, *P < *0.01; ***, *P < *0.001). (E) *NP3084-GAL4 *>* w^1118^* and *NP3084-GAL4 *>* Duox RNAi* larvae were orally infected for 30 min with S. aureus USA300 WT (10 × 10^8^ bacteria/larva). At 2 h p.i., quantitative real-time PCR analyses of the *katA* strain were performed. Transcripts levels were normalized to the corresponding *gyrB* levels. Data are means ± SEM (*n* = 3). The Mann-Whitney test was applied to compare groups (**, *P < *0.01). (F, G) *w^1118^* D. melanogaster larvae were orally infected for 30 min with WT S. aureus or the Δ*sigB*, Δ*sigB-cp-sigB*, Δ*agrC*, or Δ*agrC-cp-agrC* strain (10 × 10^8^ bacteria/larva). At 6 h p.i., quantitative real-time PCR analyses of *katA* (F), *sigB* (G, left), or *agrC* (G, right) were performed. Transcripts levels were normalized to the corresponding *gyrB* levels and expressed relative to WT results for panel F. Data are means ± SEM (*n* = 3). One-way ANOVA and then multiple-comparison tests were performed, and results were compared to those for the WT-infected group (*, *P < *0.05; **, *P < *0.01).

We first tested the virulence of Δ*sigB* and Δ*agrC* mutant strains in our model. We observed that 72% and 69% of larvae survived upon infection with the *sigB* and *agrC* mutants, respectively, at 24 h (compared to 16% of larvae surviving with the WT strain). The virulence of the Δ*sigB* and Δ*agrC* mutants was partially or totally restored when the strains were complemented with pCN57-cp-*sigB* and pCN57-cp-*agrC*, respectively (see Materials and Methods) (Fig. 4A). The attenuated virulence of the two mutants was accompanied by a reduced number of bacteria in intestines, which was also restored partially or totally when the strain was complemented with the corresponding WT allele of *sigB* and *agrC* (Fig. 4B). Partial phenotype restoration of the Δ*sigB* strain might be explained by differences in genomic and plasmid-carried *sigB* expression.

We then first assessed the specific role of SigB and Agr in *katA* transcription upon oxidative stress. For this, exponentially growing bacteria were subjected to 15 mM H_2_O_2_ in DPBS for 30 min. We observed that under nontreated conditions, *katA* expression remained unchanged in the WT, Δ*sigB*, and Δ*agrC* strains. Under H_2_O_2_ treatment, the *katA* gene underwent a higher transcription in the WT strain (16.5-fold increase) than in the Δ*sigB* and Δ*agrC* strains (4.1- and 3-fold increases) (Fig. 4C). Unexpectedly, transcriptional analyses revealed that H_2_O_2_ downregulated *sigB* expression independently from Agr (Fig. 4D, left), whereas repressive control from SigB on *agr* expression, under nontreated conditions, was lost upon H_2_O_2_ treatment (Fig. 4D, right).

Finally, we monitored *katA* gene regulation *in vivo* during larval intestinal infection. We found that S. aureus *katA* expression was dependent on intestinal H_2_O_2_ generation, as we observed a 14.7-fold decrease in expression in *Duox RNAi-*expressing larvae (*NP3084-GAL4 > Duox RNAi*) compared to that in WT larvae (*NP3084-GAL4 > w^1118^*) at 2 h p.i. (Fig. 4E). Unexpectedly, the *katA* gene was upregulated in Δ*sigB* and Δ*agrC* strains compared to the WT during intestinal infection (respectively, 25.5- and 4.7-fold increases) (Fig. 4F). Experiments performed in animals also showed that Agr repressed *sigB* expression at 6 h p.i. (Fig. 4G, left), whereas *sigB* mutation still resulted in a higher expression of *agrC* (Fig. 4G, right). All together, these results support a role for Agr as a new regulator of *katA* transcription upon H_2_O_2_ challenge, in addition to SigB and other contributors of the S. aureus oxidative-stress response.

## DISCUSSION

Here, we present an *in vivo Drosophila* larval model that allows for easy and rapid monitoring of both bacterial infection and innate host immune responses simultaneously. The use of this invertebrate model offers great potential to dissect complex host-pathogen interactions (61–63) because it has remarkable homology to mammals in innate immunity, in addition to available genetic tools and husbandry facilities.

### Intestinal infection with MRSA and MSSA clinical isolates induces larval death.

During oral infection of *Drosophila* larvae by S. aureus, the bacteria reach and establish themselves in the first half of the posterior part of the larval gut, probably due to the neutral pH specifically encountered there. This localized colonization is associated with intestine enlargement, as previously observed with the invertebrate model Caenorhabditis elegans (64). Notably, we found that infection with 10 × 10^8^ bacteria significantly killed larvae after 24 h when they were infected with the MRSA USA300 strain (ST8) or clinical isolates of methicillin-susceptible S. aureus (MSSA) with different sequence types (ST1, -5, and -30). In contrast to a previous study with non-antibiotic-resistant S. aureus strains that did not identify a bacterial killing effect in the adult stage (42), we suggest here that the killing phenotype observed in larvae is due primarily to the amount of bacteria ingested. The epidemic strain S. aureus USA300 carries an hypervirulent phenotype characterized by the expression of multiple toxins (such as the enterotoxins K and Q and the Panton-Valentine leukocidin [PVL] pore-forming toxin) and the arginine catabolic mobile element (ACME), which displays adhesive properties and improves bacterial colonization (5, 65). All these specificities may play an important role in successful intestinal establishment.

It was recently shown that larvae orally infected with Erwinia carotovora subsp. *carotovora 15* (*Ecc15*), Pseudomonas aeruginosa, or Pseudomonas entomophila are more susceptible to pathogens than adult flies infected with similar doses (66). The adult intestine undergoes basal turnover characterized by proliferation of intestinal stem cells (ISCs), which differentiate into intermediate progenitor cells named enteroblasts (EBs) and then into enterocytes (ECs) or enteroendocrine cells (EEs) (67). Upon infection with the Gram-negative pathogen *Ecc15* (68, 69) or P. entomophila (70), compensatory mechanisms, respectively, activated by the epidermal growth factor receptor (EGFR) and the JAK/STAT pathways initiate a strong mitotic response in the midgut, without modifying ISC number. This phenomenon is complementary to the intestinal antimicrobial response and essential to resist infection. Interestingly, it was also shown that the IMD pathway plays a key role in ECs shedding during infection, also favoring epithelial turnover (71). In contrast, *Drosophila* larvae do not possess ISCs and, upon *Ecc15* intestinal infection, rely on adult midgut progenitors (66). These progenitor cells differentiate into ECs; however, the authors raise the hypothesis that these cells are insufficient in number to meet the need of both intestinal repair and antimicrobial response. This may explain the particular sensitivity of the larvae to intestinal infections.

### Intestinal infection with S. aureus triggers host ROS production.

We found that S. aureus infection triggered ROS production in the gut of *Drosophila* larvae early in the infection and in a transient way. The *Drosophila* genome encodes one Duox enzyme, whereas two Duox homologs are identified in mammals (72). In flies, it was shown that the Duox enzyme could be activated by pathogen-derived uracil, unlike with commensal bacteria, which do not secrete this molecule (73). Of note, S. aureus USA300 is capable of generating uracil through pyrimidine metabolism (Kyoto Encyclopedia of Genes and Genomes pathway). We herein showed that live bacteria, as well as heat-killed bacteria, were able to increase *Duox* gene transcription, suggesting that both the S. aureus cell wall and secreted factors play a role in this mechanism. We confirmed Duox activation by measuring an increase in ROS generation, occurring in the first hours of infection. These data somewhat contradict an earlier work done by Hori et al. (42) reporting that S. aureus *Drosophila* feeding did not induce ROS production. This apparent discrepancy is likely due to the ROS quantitation method used in both studies. To quantify the ROS amount, Hori and colleagues used hydro-Cy3, a compound for which measurement may be influenced by mitochondrial membrane potential (74), which was shown to be modified during bacterial infection (75). This discrepancy may also be due to the method used to dissect the larval intestine. In insects, Malpighian tubules play a key role in detoxification and hemolymph filtering (as with the kidneys and liver in mammals). They are intimately linked to the stress status of the fly (76), and it was recently shown that Malpighian tubules play an active role during oral infection by sequestering excessive ROS and oxidized lipids (54). Including them during dissection might greatly affect results by hiding the specific intestinal ROS signal. In another model of orally infected black soldier flies, S. aureus was shown to be able to induce *Duox* gene expression, as well as to increase the H_2_O_2_ concentration, in a short time frame (77). Overall, this work confirms the importance of generating intestinal oxidative stress to clear colonizing pathogens as well as the necessity for the bacterium to acquire efficient oxidative-stress-resistant systems. Our results demonstrate that the S. aureus USA300 *katA* gene is necessary to increase bacterial virulence *in vivo* and to assess its colonization capacities. Of note, the importance of the S. aureus catalase gene has previously been shown *in vitro* during intracellular infection in murine macrophages or *in vivo* through intraperitoneal injection with a Δ*katA* clinical bovine strain (78).

**FIG 5:**
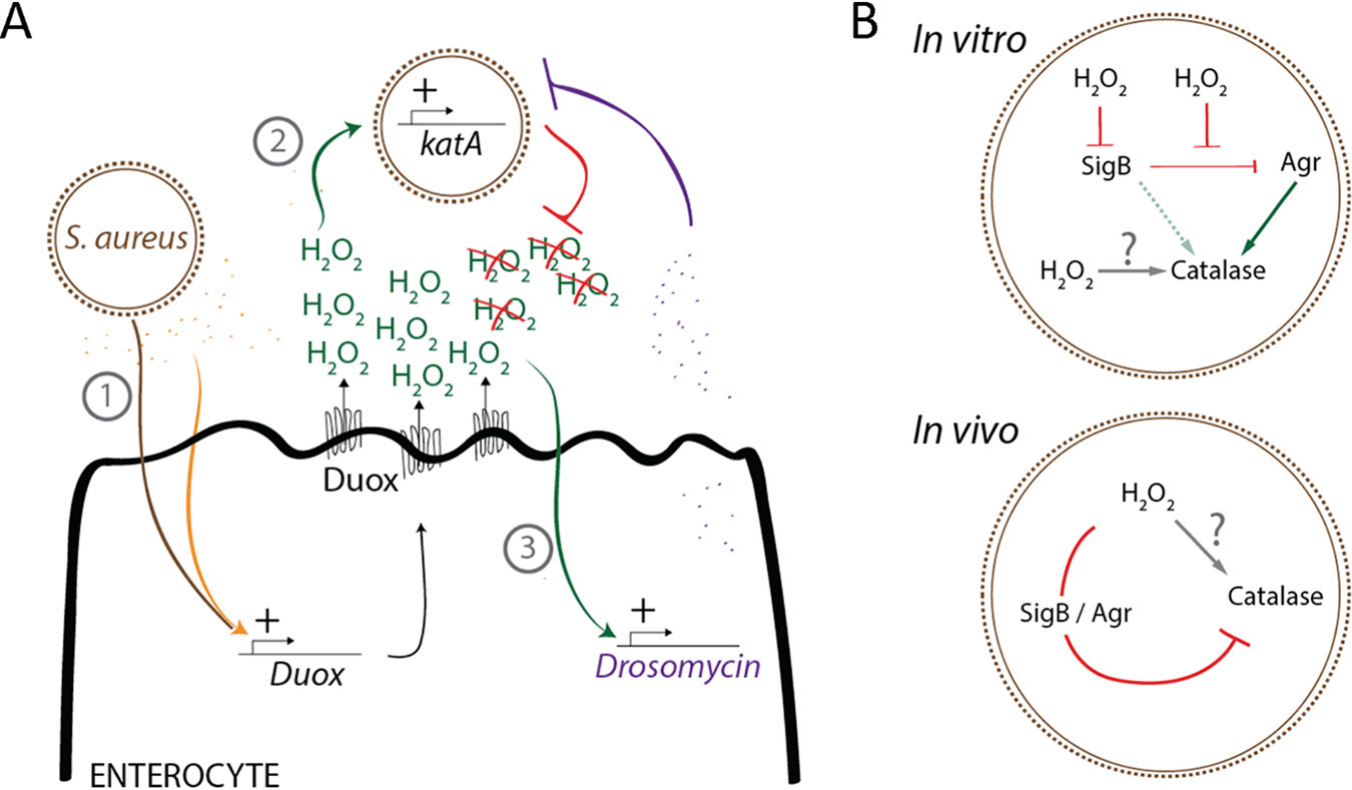
Model of S. aureus USA300 H_2_O_2_ quenching in *Drosophila* intestine. (A) In the intestine, S. aureus exerts a cell wall and secretome-mediated activation of *Duox* transcription, thus leading to H_2_O_2_ production in the gut lumen (1). This activates bacterial *kata* transcription (2), which will quench hydrogen peroxide molecules in order to both counteract bacterial clearing from ROS and limit *Drosomycin* gene transcription, whose expression is positively regulated by H_2_O_2_ (3). (B) *In vitro*, under H_2_O_2_ treatment, S. aureus *katA* expression is activated by Agr and to a lesser extent by SigB, which undergoes negative transcriptional regulation by H_2_O_2_. Additional regulators positively influence *katA*. *In vivo*, we observed that SigB and Agr repress *katA* expression, thus highlighting the complex regulatory network that influences *katA* expression.

Our work has demonstrated the link between ROS production and activation of Toll signaling in the guts of *Drosophila* larvae after exposure to S. aureus USA300 (Fig. 5). Presumably, this mechanism potentiates the host immune response against harmful pathogens, such as S. aureus. The established link between ROS and Toll pathway initiation in *Drosophila* was established previously, and it was also shown that *Wolbachia*-infected mosquitoes exhibited an increase in *Duox2* transcription that was sufficient to induce transcription of the Toll pathway-sensitive AMPs cecropins and defensins (79). Interestingly, research in mammals suggests that ROS may alter the activity of the iκB kinase (IKK) complex in the cytoplasm or the DNA-binding capacity of NF-κB in the nucleus. (80). These observations highlight the need for bacteria to consistently control the host clearance strategy by simultaneously acting on the immune response and the ROS pool.

### *katA* undergoes different regulatory networks *in vitro* and *in vivo*.

The present work also shed light on the role of SigB and Agr in the expression of S. aureus *katA in vitro* or *in vivo* (Fig. 5). We first observed *in vitro* that SigB and Agr did not influence *katA* transcription under the nontreated condition and showed that, upon H_2_O_2_ treatment, SigB upregulated *katA* expression. These data are in agreement with previous observations reporting the increased susceptibility of a Δ*sigB* mutant (Newman and MSSA backgrounds) to H_2_O_2_ treatment (81, 82). However, we have also shown that SigB played a minor contribution to the regulation of *katA* because, in the presence of H_2_O_2_, *sigB* expression was reduced and its negative influence on Agr expression (83) was repressed. Hence, we propose here that Agr is an important new intermediate in *katA* upregulation.

*In vivo*, S. aureus can undergo multiple intestinal stresses, such as pH changes, iron sequestration (84), interactions with microbiota (85), antibiotic treatments (86), and oxygen or nutrient limitations (87). Somewhat unexpectedly, we found that the *katA* gene was upregulated in *sigB* and *agrC* mutants, suggesting a minor role for these master regulators in its *in vivo* regulation and implying the contribution of multiple additional cues. Notably, several infection models suggest that SigB was not important for bacterial virulence in the mouse abscess model (88), osteomyelitis, pyelonephritis (89), or nematode digestive infection (90). Thus, one can speculate that in this complex environment, resistance to oxidative stress, despite a clear role in limiting bacterial clearance, may also allow the acquisition of a fitness advantage.

Finally, we reported that intestinal infections with *S*. Typhimurium and S. flexneri induced death in *Drosophila* larvae and that this was associated with an antimicrobial response consisting of the IMD pathway and ROS production. We therefore propose that this infection model can serve as an easily manipulated alternative to mammalian models to study innate host immune responses triggered during infection with human bacterial pathogens.

## MATERIALS AND METHODS

### Bacterial strains.

The epidemic clone S. aureus USA300-LAC (designated S. aureus USA300 WT) as well as its isogenic derivatives were provided by Biodefense and Emerging Infections Research Resources (BEI Resources). S. aureus growth was performed in brain heart infusion (BHI) broth or agar or chromID MRSA agar at 37°C. Micrococcus luteus was grown in Luria-Bertani (LB) broth at 30°C. Salmonella Typhimurium SL1344 was grown in LB broth or agar at 37°C, and Shigella flexneri was grown in tryptic soy broth or agar (TSA) supplemented with Congo red dye (final concentration, 0.01%) to induce a type 3 secretion system (T3SS)-dependent secretion of virulence factors (91). Only pigmented colonies from TSA plates were used to prepare liquid cultures. pRN11 (48)- and pCN57 (92)-carrying strains were transformed by electroporation, with the following settings: 2,450 V, 100 Ω, 25 μF, and a time constant of 2.3 to 2.5 ms.

For heat-killed bacteria, overnight-grown cultures were centrifuged, the supernatant was removed, and bacteria were resuspended in DPBS. Bacteria were heated at 80°C for 20 min and further used for infection.

For complemented strains, we used the pCN57 shuttle vector. For the pCN57-cp-*katA* plasmid, the PCR product of the pCN57 vector (treated with restriction enzyme DpnI to eliminate the template plasmid) was fused to the *catalase* gene amplicon, followed by a kanamycin resistance cassette (kanamycin resistance gene *nptII* fused with the *pGro* promoter; 1,111 bp in total). All PCR products were assembled with the HiFi DNA assembly master mix kit. Similarly, for the pCN57-cp-*agrC* plasmid, the three PCR products (pCN57, *agrC*, and *kan* cassette) were purified and fused with the HiFi DNA assembly master mix. For the pCN57-cp-*sigB* plasmid, we used pCN57-cp-*agrC* as the template to amplify the pCN57 vector with the kanamycin cassette. This PCR product (after treatment with the restriction enzyme DpnI) was fused to the *sigB* PCR product using the HiFi DNA assembly master mix kit.

All strains and plasmids are defined in Table 1.

**TABLE 1:** Strains and plasmids used in this study

Strain or plasmid	Relevant characteristics	Source or reference(s)
Strains		
Staphylococcus aureus USA300 WT	MRSA, USA300-LAC (ST8), Ery^r^	BEI Resources (NR46070)
Staphylococcus aureus USA300 WT/pRN11 (mCherry)	S. aureus USA300 WT complemented with the pRN11 plasmid, Cm^r^, mCherry expressing	This work
Staphylococcus aureus USA300 Δ*katA*	MRSA USA300 JE2 isolate (plasmid-cured derivative of strain USA300-LAC, ST8), Ery^r^, transposon insertion in the *catalase* gene, position 1350703, reverse orientation	BEI Resources (NE1366)
Staphylococcus aureus USA300 Δ*katA/*pRN11 (mCherry)	S. aureus Δ*katA*, complemented with the pRN11 plasmid, Cm^r^, mCherry expressing	This work
Staphylococcus aureus USA300 Δ*katA*-cp-*kat*	Complemented strain JE2 Δ*katA* containing pCN57-cp-*kat*, Ery^r^ Kan^r^	This work
Staphylococcus aureus USA300 Δ*sodA*	MRSA USA300 JE2 isolate (plasmid-cured derivative of strain USA300-LAC, ST8), Ery^r^, transposon insertion in the *superoxide dismutase A* gene, position 154256, reverse orientation	BEI Resources (NE1224)
Staphylococcus aureus USA300 Δ*sodA/*pRN11 (mCherry)	S. aureus Δ*sodA*, transformed with the pRN11 plasmid, Cm^r^, mCherry expressing	This work
Staphylococcus aureus USA300 Δ*sodM*	MRSA USA300 JE2 isolate (plasmid-cured derivative of strain USA300-LAC, ST8), Ery^r^, transposon insertion in the *superoxide dismutase M* gene, position 1664049, reverse orientation	BEI Resources (NE1932)
Staphylococcus aureus USA300 Δ*sodM*/pRN11 (mCherry)	S. aureus Δ*sodM*, transformed with the pRN11 plasmid, Cm^r^, mCherry expressing	This work
Staphylococcus aureus USA300 Δ*gpxA1*	MRSA, USA300 JE2 isolate (plasmid-cured derivative of strain USA300-LAC, ST8), Ery^r^, transposon insertion in the *glutathione peroxidase A1* gene, position 1319250, reverse orientation	BEI Resources (NE1730)
Staphylococcus aureus USA300 Δ*gpxA1*/pRN11 (mCherry)	S. aureus Δ*gpxA1*, transformed with the pRN11 plasmid, Cm^r^, mCherry expressing	This work
Staphylococcus aureus USA300 Δ*gpxA2*	MRSA, USA300 JE2 isolate (plasmid-cured derivative of strain USA300-LAC, ST8), Ery^r^, transposon insertion in the *glutathione peroxidase A2* gene, position 2764669, reverse orientation	BEI Resources (NE1366)
Staphylococcus aureus USA300 Δ*gpxA2*/pRN11 (mCherry)	S. aureus Δ*gpxA2*, transformed with the pRN11 plasmid, Cm^r^, mCherry expressing	This work
Staphylococcus aureus USA300 S*igB*	MRSA, USA300 JE2 isolate (plasmid-cured derivative of strain USA300-LAC, ST8), Ery^r^, transposon insertion in *rpoF* (*sigmaB*) gene, position 2185624, reverse orientation	BEI Resources (NE1366)
Staphylococcus aureus USA300 Δ*sigB/*pRN11 (mCherry)	S. aureus Δ*sigB*, transformed with the pRN11 plasmid, Cm^r^, mCherry expressing	This work
Staphylococcus aureus USA300 Δ*sigB*-cp-*sigB*	Complemented strain S. aureus JE2 Δ*sigB* containing pCN57-cp-*sigB*, Ery^r^ Kan^r^	This work
Staphylococcus aureus USA300 Δ*agrC*	MRSA, USA300 JE2 isolate (plasmid-cured derivative of strain USA300-LAC, ST8), Ery^r^, transposon insertion in the *accessory gene regulator protein C* gene, position 2147768, reverse orientation	BEI Resources (NE1366)
Staphylococcus aureus USA300 Δ*agrC*/pRN11 (mCherry)	S. aureus Δ*agrC*, transformed with the pRN11 plasmid, Cm^r^, mCherry expressing	This work
Staphylococcus aureus USA300 Δ*agrC*-cp-*agrC*	Complemented strain S. aureus JE2 Δ*agrC* containing pCN57-cp-*agrC*, Ery^r^ Kan^r^	This work
Staphylococcus aureus P1	Clinical isolate MSSA ST1, PVL^+^, isolated from blood culture	98
Staphylococcus aureus P2	MSSA ST5, PVL^–^, isolated from blood culture	This work
Staphylococcus aureus P3	MSSA ST30, PVL^–^, isolated from a skin infection	This work
Micrococcus luteus		Gift from D. Ferrandon laboratory
Salmonella Typhimurium/pGG2 DsRed	Salmonella enterica serovar Typhimurium SL1344, transformed with pGG2 expressing DsRed under the control of the *rpsM* promoter, Amp^r^	99
Shigella flexneri/pMW211 pDsRed	Shigella flexneri strain M90T Sm (serotype 5a), DsRed expressing, Amp^r^	100, 101

Plasmids		
pRN11	*sarA* p1-mCherry, vector backbone pCM29 in E. coli DC10b, Amp^r^ in E. coli and Cm^r^ in Gram-positive bacteria	48
pCN57	Plasmid containing the *gfp* cassette (constitutive expression), Ery^r^	92
pCN57-cp-*katA*	pCN57 plasmid containing the *catalase* gene, with 300 bp upstream of the gene, followed by a kanamycin resistance gene, Kan^r^	This work
pCN57-cp-*agrC*	pCN57 plasmid containing the *agrC* gene with 162 bp upstream of the gene followed by a kanamycin resistance cassette, Kan^r^	This work
pCN57-cp-*sigB*	pCN57 plasmid containing the *sigB* gene, with 131 bp upstream of the gene, followed by a kanamycin resistance cassette, Kan^r^	This work

### Whole-genome sequencing and analysis.

Genomic DNA was extracted using a DNeasy blood and tissue kit. Genomic libraries were prepared using a Nextera XT kit and then multiplexed and sequenced on an Illumina MiniSeq instrument (2× 150 paired-end reads).

Single-nucleotide polymorphisms (SNPs) and small indels were assessed using Snippy v3.1 (https://github.com/tseemann/snippy). Briefly, Snippy was used to map the raw short reads against the annotated assembly of the parental strain (Staphylococcus aureus strain JE2; GenBank accession no. CP020619.1).

Assemblies were collected with a Unicycler assembler, available through PATRIC version 3.6.8 (93, 94). Prokka version 1.14.0 (95) was used to annotate the assemblies by allowing the verification of the transposon insertion site.

### Drosophila stocks and rearing.

Drosophila melanogaster was maintained on a fresh medium prepared with the Nutri-fly Bloomington formulation (Genesee Scientific, San Diego, CA, USA), supplemented with 64 mM propionic acid. *N*-Acetyl-l-cysteine (NAC)-supplemented medium was prepared at the final concentration of 1 mM (A9165; Sigma) (96).

All *Drosophila* stocks are defined in Table 2.

**TABLE 2:** D. melanogaster lines

Drosophila melanogaster line	Source	Reference or identifier
*w^1118^* (control line)	D. Ferrandon	
*ywDD1*;; (control line)	D. Ferrandon	102
*yw drs-GFP dipt-Lac*Z;;*spz^rm7^/TM6c* (*ywDD1 + spz^rm7^/TM6c*)	D. Ferrandon	102
*w NP3084-GAL4^+^*	W. J. Lee	DGRC (113094)
*w UAS-Duox RNAi/CYO*	W. J. Lee	53

### Infection experiments.

Oral infections were performed on mid-L3 larvae (3.5 days after egg laying). For each test, animals were placed in a 2-ml microcentrifuge tube filled with 200 μl of crushed banana and 200 μl of overnight bacterial culture for 30 min. Bacterial infectious doses were adjusted by measuring culture turbidity at an optical density at 600 nm (OD_600_; considering that an OD_600_ of 1 is 5.10^8^ bacteria/ml). Animals were blocked by a foam plug to be sure that they remained in the bottom of the tube for the whole infection time. After 30 min, they are washed briefly in 30% ethanol and placed in a petri dish with fresh fly medium without yeast. Infections and waiting times were performed at 29°C. Larvae were dissected at the indicated time points for reverse transcription-quantitative PCR (RT-qPCR) analyses, bacterial counts, and ROS quantification.

For oral infection of adult flies, 5- to 7-day-old adults were starved for 2 h in empty vials at 25°C. After starvation, flies were flipped into an infection vial with medium and completely covered with a Whatman paper disk. The disk was soaked with 100 μl of a 5% sucrose solution supplemented or not with bacteria at the indicated infectious doses. After 30 min of oral infection, flies were flipped to fresh fly medium without yeast (changed every day).

### CFU counts.

At the indicated time points, larvae were dissected (at least 10 animals per point) and guts homogenized in 400 μl of DPBS (Gibco, ThermoFisher Scientific, MA, USA) with a Mikro-Dismembrator S (Sartorius Stedim, Aubagne, France). Samples were serially diluted and plated on BHI agar (with antibiotics, when used). For CFU counts from hemolymph, animals were briefly washed in 70% ethanol, rinsed in sterile DPBS, and bled into a 200-μl DPBS drop on the slide. Samples were directly plated on BHI agar plates for S. aureus counts or LB agar for M. luteus.

### pH survival assay.

Assays were performed in BHI agar with an adapted pH. pH was adjusted with sodium hydroxide or hydrochloric acid solutions at the selected conditions: pHs of 3, 5, 7, 9, and 11. Media adjusted at pHs 3 and 5 were buffered with 2-(*N*-morpholino)-ethane sulfonic acid (MES; 30 mM; Euromedex, Souffelweyersheim, France), at pH 7 with Tris-(hydroxymethyl) ammonium (Tris, 30 mM; Merck), and at pHs 9 and 11 with 2-[*N*-cyclohexylamino]ethane-sulfonic acid (CHES; 30 mM; Sigma-Aldrich). Fresh bacterial cultures that reached an OD_600_ of 0.3 to 0.6 were washed one time with PBS and then diluted in the different buffers to reach the concentration of 2 × 10^7^ bacteria/ml. At the indicated time points, 50 μl from each culture was sampled, serially diluted, and plated on BHI agar.

### ROS quantification and visualization. (i) ROS quantification.

The amount of ROS in dissected guts (from 10 animals) was estimated using 2′,7′-dichlorodihydrofluorescein diacetate (H_2_DCFDA; C6827; ThermoFisher Scientific, MA, USA) by following the manufacturer's instructions. For larval gut dissection, we carefully removed the Malpighian tubules, as they can strongly influence ROS level, and then tissues were homogenized in H_2_DCFDA mix. Fluorescence was measured 30 min after the mix preparation in a Berthold TriStar LB941 multiplate reader (Berthold France SAS, Thoiry, France). Results were normalized to those for total protein for each sample. The protein concentration was quantified using the Pierce bicinchoninic acid (BCA) colorimetric assay (Life Technologies, Ca, USA) by following the manufacturer’s instructions.

### (ii) ROS visualization.

Guts were dissected at the indicated times on glass slides, incubated in H_2_DCFDA (10 μM) for 15 min, and live imaged with a Zeiss Axio Imager Z2 Apotome microscope.

### Larval imaging. (i) Whole-gut stainings.

Guts were dissected in PBS, fixed for at least 1 h at room temperature in 4% paraformaldehyde in PBS, and permeabilized in PBS plus 0.1% Triton X-100 for 30 min. They were stained with BODIPY 493/503 at a 1/100 dilution (D3922; ThermoFisher Scientific, MA, USA) for 1 h, stained with DAPI (4′,6-diamidino-2-phenylindole) at the dilution 1.43 μM for 10 min, washed with PBS, and mounted in Mowiol 4-88 (17951-500; BioValley, France).

### (ii) LSFM.

For sample preparation, animals were first fixed in ScaleCUBIC-1 (reagent 1) for at least 4 days and cleared in ScaleCUBIC-2 (reagent 2) for at least 2 days according to the method of Susaki et al. (97). Briefly, to prepare 500 g of reagent 1 solution, 125 g of urea and 156 g of 80% (by weight) Quadrol were dissolved in 144 g of distilled water (dH_2_O). After complete dissolution under agitation, we added 75 g of Triton X-100 and then degassed the reagent with a vacuum desiccator (∼0.1 MPa, ∼30 min) (97). Then, samples were cleared with ScaleCUBIC-2 (reagent 2). To perform the Lightsheet fluorescence microscopy (LSFM) imaging, samples were embedded in 4% low-melting-point agarose (ThermoFisher Scientific, France) dissolved in R2 medium by using a glass cylindrical capillary, and we allowed embedding overnight. Images were acquired with a Lightsheet Z.1 microscope (Carl Zeiss, Germany) equipped with a Plan-Apochromat 20×/NA1 R2-immersion lens objective with left and right illumination.

### RT-qPCR.

For mRNA extraction, dissected guts were collected at the indicated time points and homogenized with a Mikro-Dismembrator S (Sartorius Stedim, Aubagne, France). Total RNA was isolated using TRIzol reagent and dissolved in RNase-free water. Five hundred nanograms of total RNA was then reverse transcribed in a 20-μl reaction volume using the LunaScript RT supermix kit (E3010; New England Biolabs, MA, USA). Quantitative PCR was performed by transferring 2 μl of the RT mix to the qPCR mix prepared with Luna Universal qPCR master mix (M3003; New England Biolabs, MA, USA) according to the manufacturer’s recommendations. All the primers used for this experiment are defined in Table 3, and their amplification efficiency was checked before any further analysis. Reactions were performed on a 7900HT Fast real-time PCR system (Applied Biosystems) according to the standard settings of the system software. The thermal cycling conditions were as follows: an initial denaturation at 95°C for 1 min, followed by 40 cycles of 95°C for 15 s and 60°C for 30 s. We used relative quantification with normalization against the reference gene *RP49.*

**TABLE 3:** Oligonucleotides used in this study

Oligonucleotide	Sequence^*a*^
*RP49* F	GAC GCT TCA AGG GAC AGT ATC TG
*RP49* R	AAA CGC GGT TCT GCA TGA G
*Drosomycin* F	CGT GAG AAC CTT TTC CAA TAT GAT
*Drosomycin* R	TCC CAG GAC CAC CAG CAT
*Diptericin* F	GCT GCG CAA TCG CTT CTA CT
*Diptericin* R	TGG TGG AGT GGG CTT CAT G
*Duox* F	CAA CAC CAC GGG ATG TCG AA
*Duox* R	CGA CCA TCA GCT GCT CCA TT
*katA* F	CTG GGA TTT CTG GAC GGG TC
*katA* R	TGA GAA CCG AAC CCA TGC AT
*gyrB* F	AGG TGG TAC GCA TGA AGA CG
*gyrB* R	TTC AAC CAC TGT ACG TGC GA
*rpoF (sigB)* F	TGC GTT AAG TGT TGA TCA TTC CA
*rpoF (sigB)* F	TGG TCA TCT TGT TGC CCC AT
*agrC* F	ACC CTA TCA TTC GCG TTG CA
*agrC* R	CGT GGT ATA TCA TCA GCG CA
*pCN57 katA* F	atc gga ggg ttt att ctg caC AGT AGC TAC AAA TAG ACC
*pCN57 katA* R	atc cat aca aTT ATT TTT CAA AGT TTT CGT ATG TTT C
*pCN57 kana* F	tga aaa ata aTT GTA TGG ATT AGT CGA GC
*pCN57 kana* R	ggg atc ctc tag agt cga ccT CAG AAG AAC TCG TCA AG
*pCN57-cp-agrC F*	gtt ctt ctg aGG CGC GCC TAT TCT AAA TG
*pCN57-cp-agrC R*	tta cc aat gtT CTT AAA TTA ATT AGT TAA CGA ATT CGA GC
*agrC* F	taa ttt aag aAC ATT GGT AAC ATC GCA G
*agrC* R	atc cat aca aAT CCT TAT GGC TAG TTG TTA ATA ATT TC
*Kana (agrC)* F	cca taa gga tTT GTA TGG ATT AGT CGA GC
*Kana (agrC)* R	tag gcg cgc cTC AGA AGA ACT CGT CAA G
*pCN57-cp-sigB* F	aat ttg ttt aTT GTA TGG ATT AGT CGA GC
*pCN57-cp-sigB* R	taa aaa gtc tTG CAG AAT AAA CCC TCC G
*sigB* F	tta ttc tgc aAG ACT TTT TAC GCG AAG G
*sigB* R	atc cat aca aTA AAC AAA TTC TAT TGA TGT GC

a Lowercase and uppercase letters indicate the pairings at each sequence.

### Statistical analysis.

Data are represented as means ± standard errors of the means (SEM). Statistical tests were performed with GraphPad (Prism 6). For experiments with two groups of samples, the Mann-Whitney test was performed. For experiments with different conditions and groups, we applied one-way or two-way analysis of variance (ANOVA). For survival curves, results from 3 independent experiments were grouped (at least 60 animals) and analyzed by the Kaplan-Meier test. For RT-qPCRs, at least 10 animals were included per point (*, *P < *0.05; **, *P < *0.01; ***, *P < *0.001).

### Data availability.

The sequences reported in this paper are available in NCBI’s BioProject database under accession no. PRJNA701878.
