# Supplementary figures and images for: Reactive Oxygen Species-Dependent Innate Immune Mechanisms Control Methicillin-Resistant Staphylococcus aureus Virulence in the Drosophila Larval Model
Source: mBio. 2021 Jun 15;12(3):e00276-21. doi: 10.1128/mBio.00276-21 (PMC8262968; doi:10.1128/mBio.00276-21)

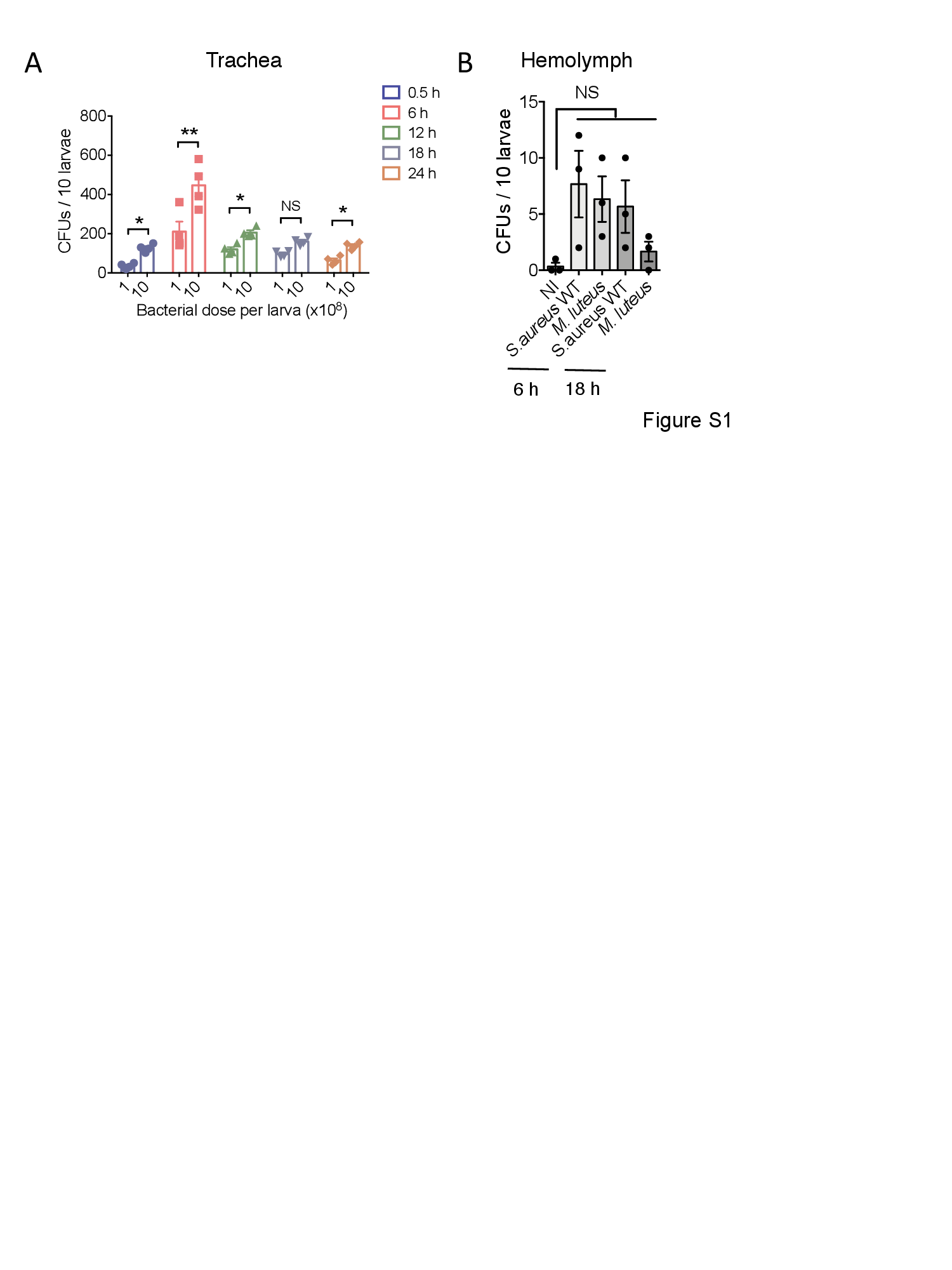

Supplement: FIG S1 [file mbio.00276-21-sf001.tif]

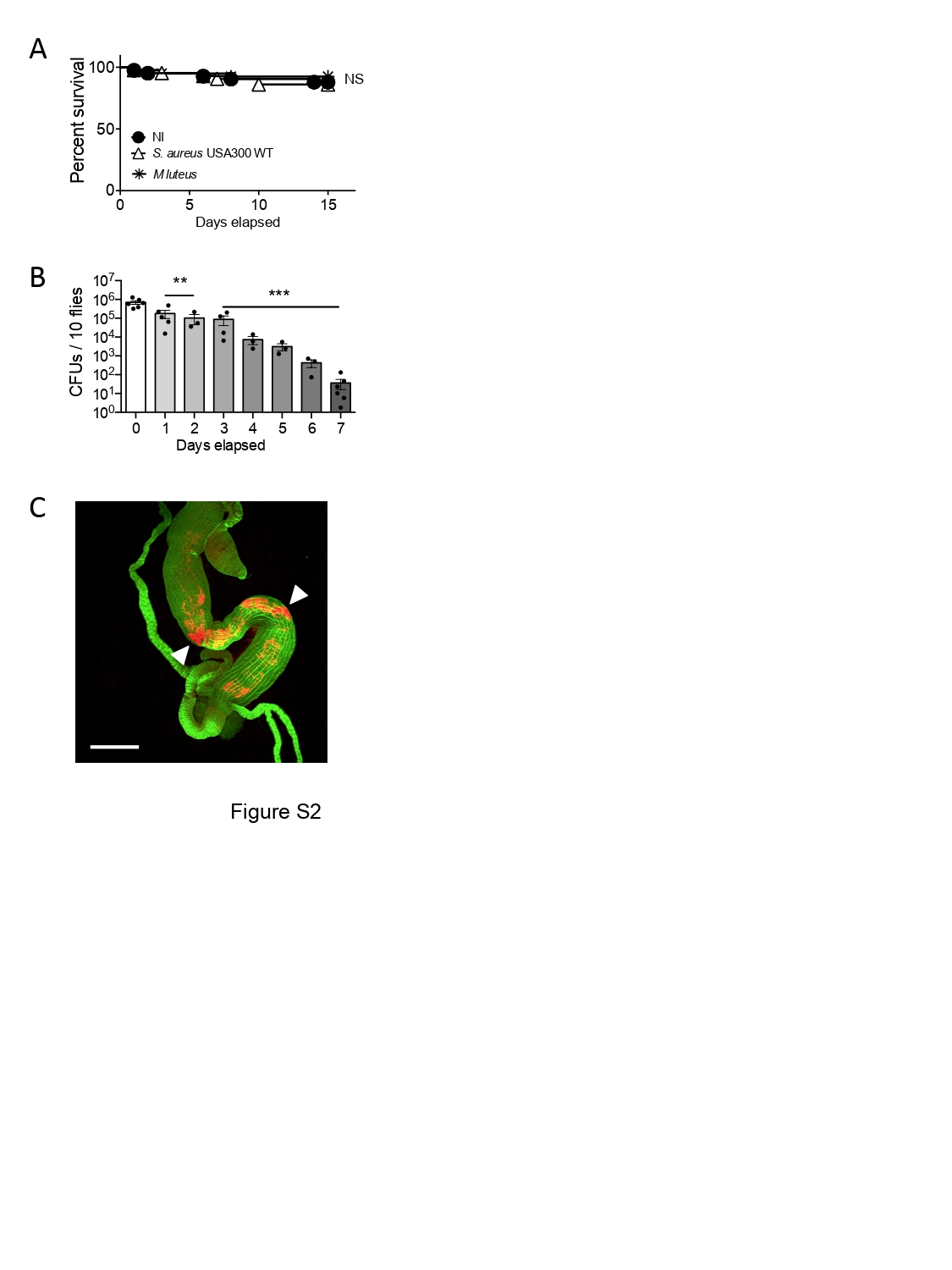

Supplement: FIG S2 [file mbio.00276-21-sf002.tif]

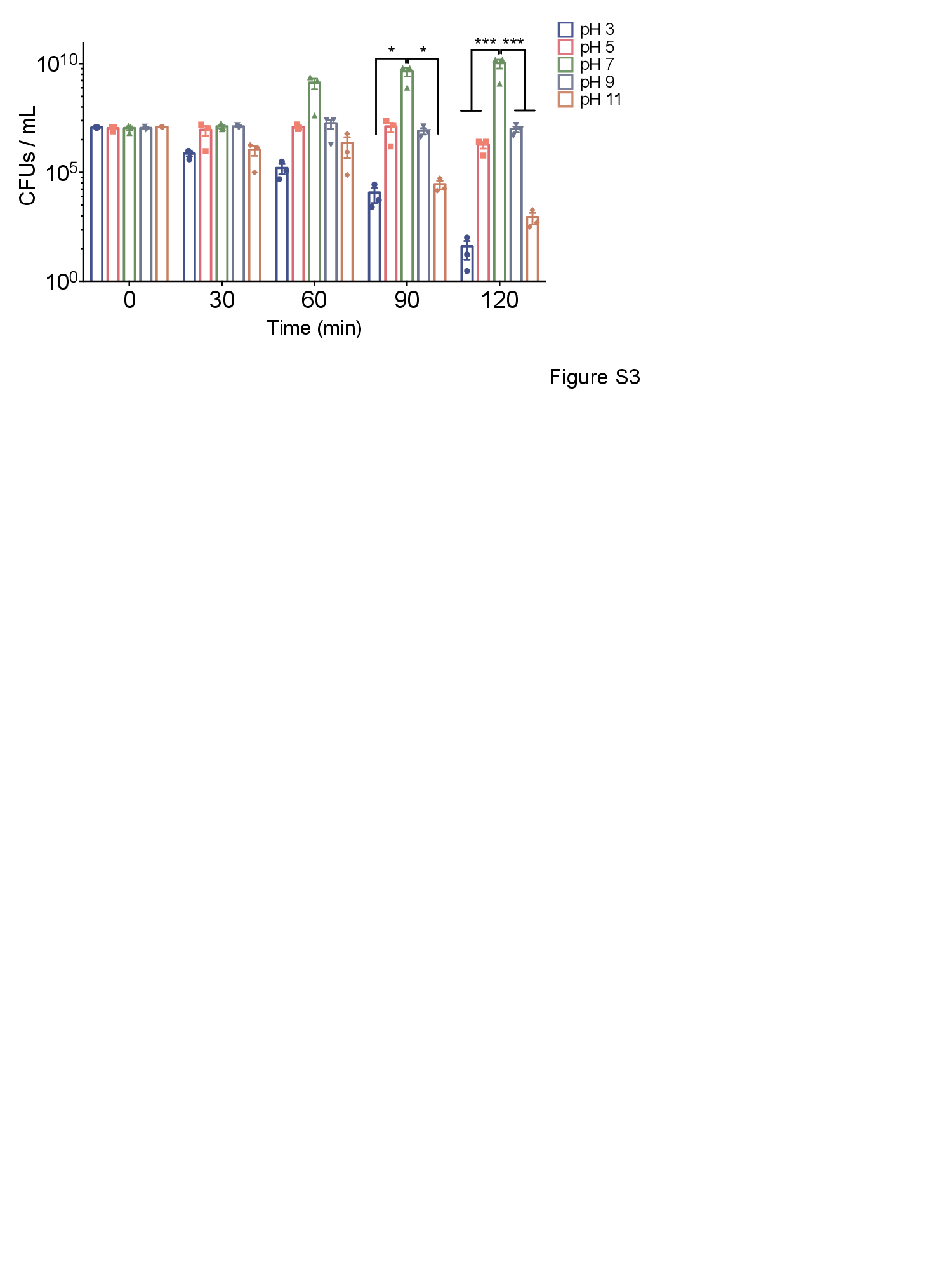

Supplement: FIG S3 [file mbio.00276-21-sf003.tif]

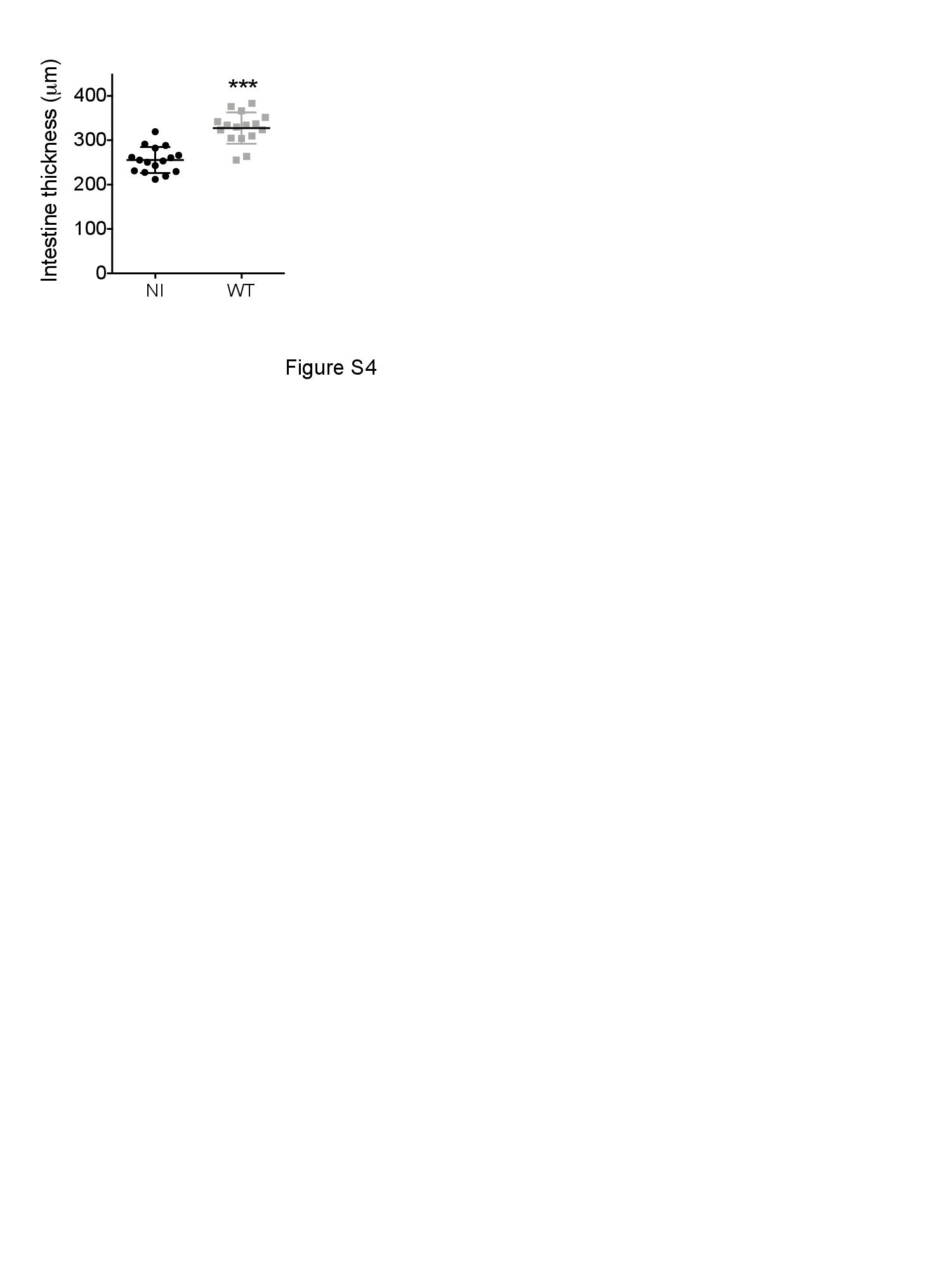

Supplement: FIG S4 [file mbio.00276-21-sf004.tif]

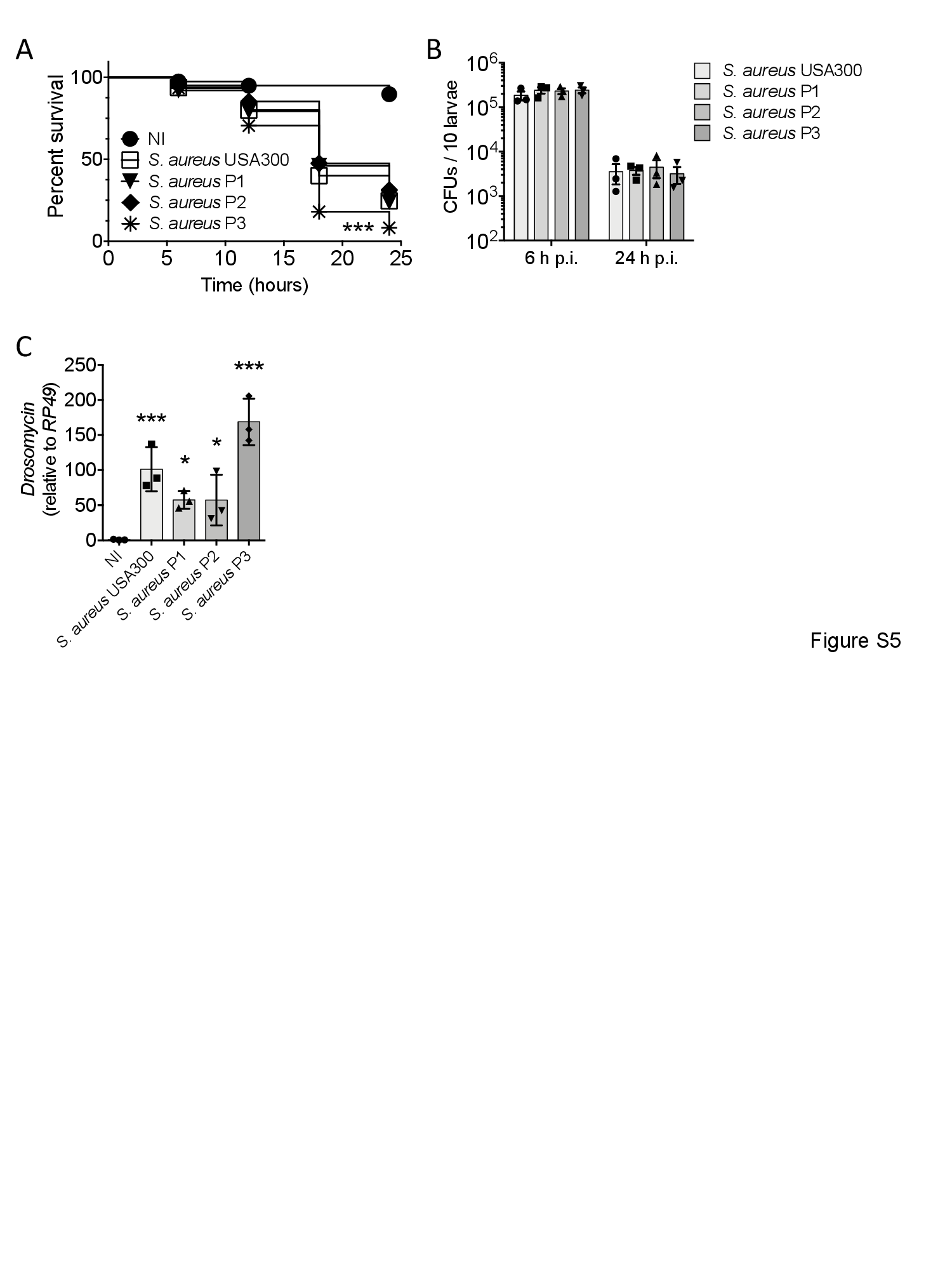

Supplement: FIG S5 [file mbio.00276-21-sf005.tif]

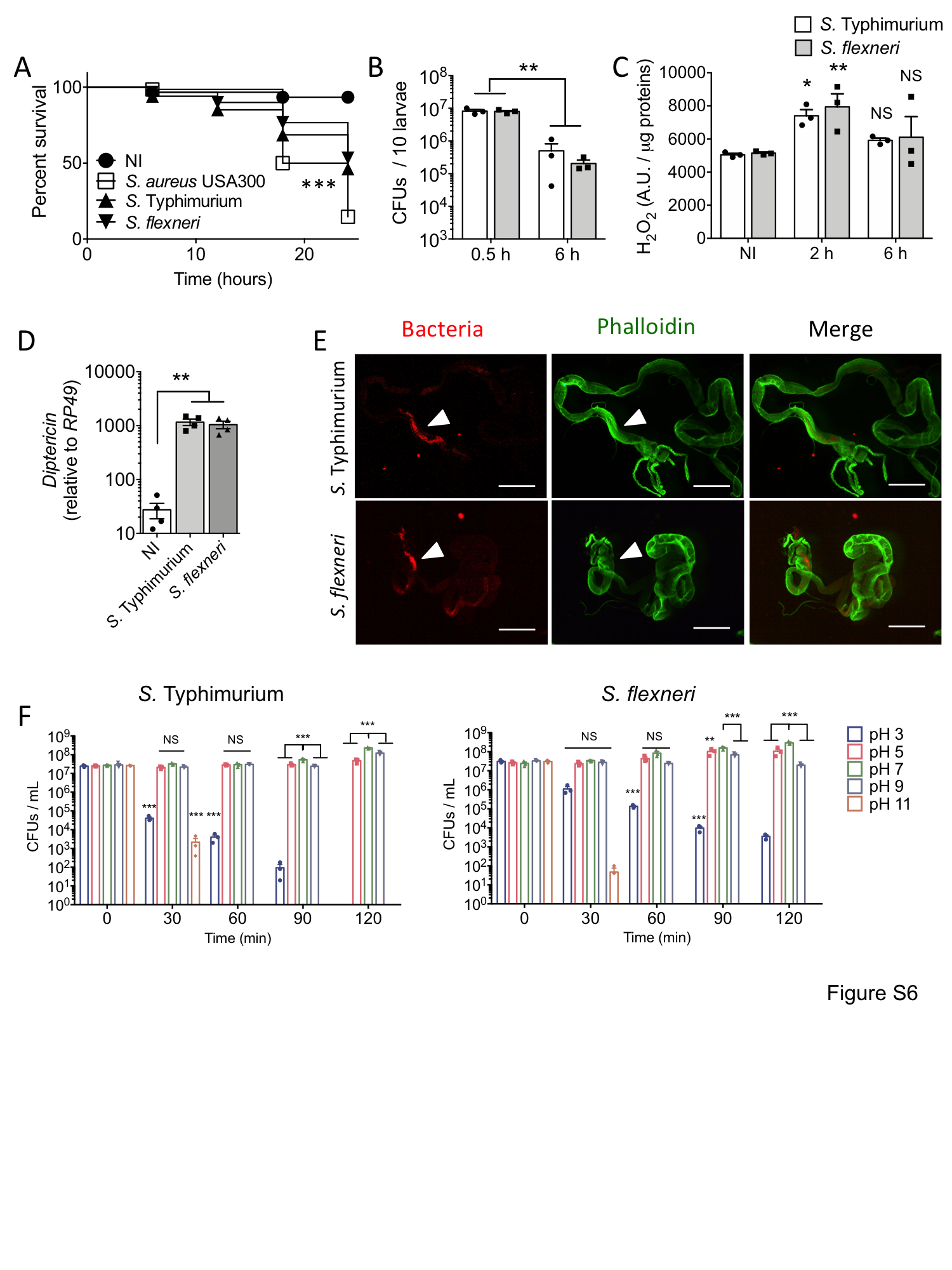

Supplement: FIG S6 [file mbio.00276-21-sf006.tif]

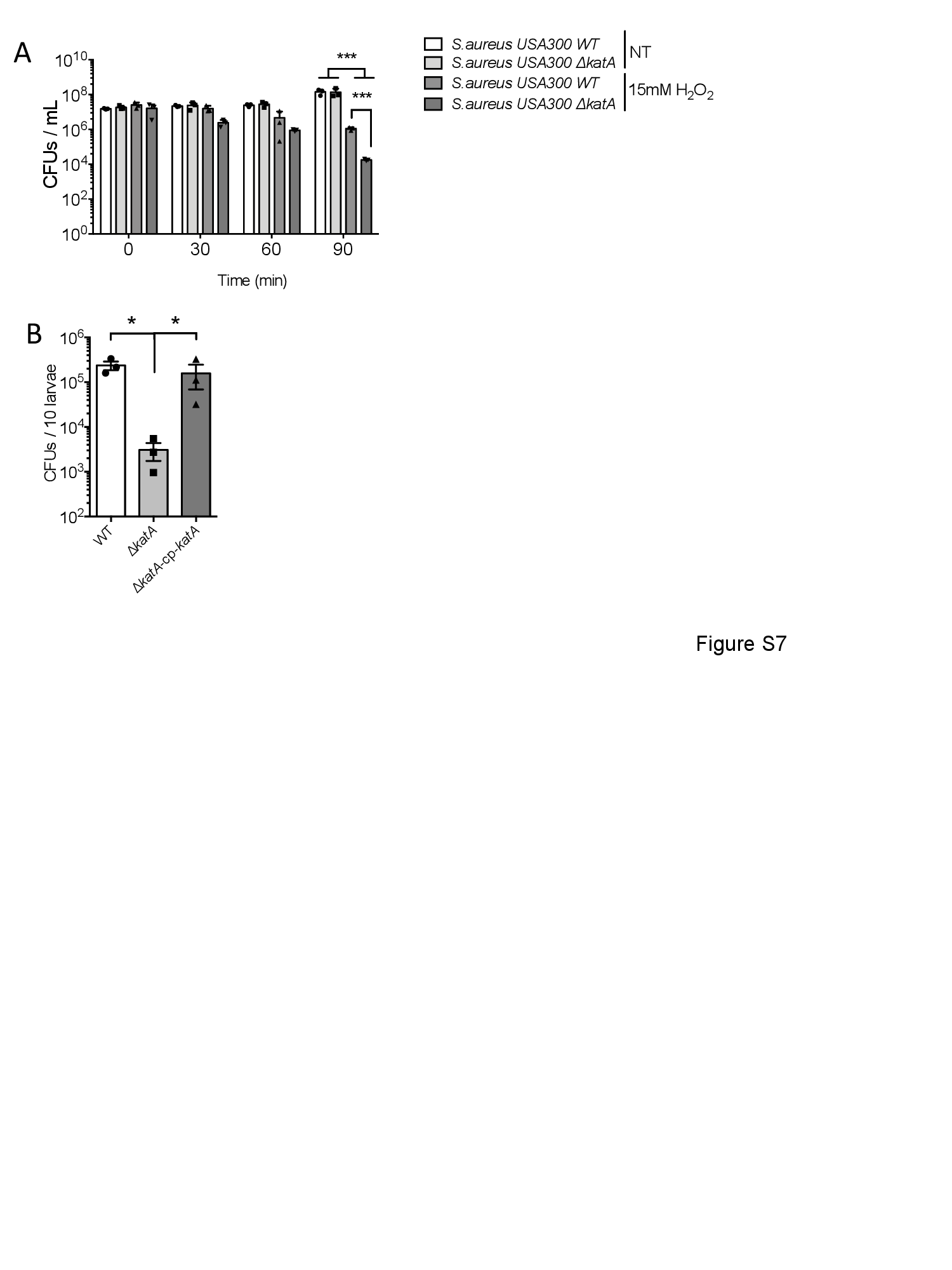

Supplement: FIG S7 [file mbio.00276-21-sf007.tif]
